# Multi-omic Analysis of Human B-cell Activation Reveals a Key Lysosomal BCAT1 Role in mTOR Hyperactivation by B-cell receptor and TLR9

**DOI:** 10.21203/rs.3.rs-4413958/v1

**Published:** 2024-05-30

**Authors:** Benjamin Gewurz, Rui Guo, Matthew Lim, Hardik Shah, Joao Paulo, Yuchen Zhang, Haopeng Yang, Liang Wei Wang, Daniel Strebinger, Nicolas Smith, Meng Li, Merrin Leong, Michael Lutchenkov, Jin-Hua Liang, Zhixuan Li, Yin Wang, Rishi Puri, Ari Melnick, Michael Green, John Asara, Adonia Papathanassiu, Steven Gygi, Vamsi Mootha

**Affiliations:** Brigham and Women’s Hospital; Tufts University; Department of Cell Biology, Harvard Medical School; The University of Chicago; Harvard Medical School; Brigham and Women’s Hospital; Department of Lymphoma/Myeloma, University of Texas MD Anderson Cancer Center; Agency for Science, Technology and Research (A*STAR); Broad Institute; Brigham and Women’s Hospital; Department of Medicine, Division of Hematology & Medical Oncology, Weill Cornell Medicine; Brigham and Women’s Hospital; Brigham and Women’s Hospital; Brigham and Women’s Hospital; Tufts University; Brigham and Women’s Hospital; Department of Biomedical Sciences, College of Veterinary Medicine, Cornell University; Weill Cornell Medicine; Department of Lymphoma/Myeloma, University of Texas MD Anderson Cancer Center; Department of Pathology; Ergon Pharmaceuticals, LLC, P.O. Box 1001; Harvard University; Massachusetts General Hospital / HHMI

**Keywords:** immunometabolism, branched chain amino acid metabolism, mTOR, reductive stress, branched chain ketoacid, immune checkpoint, pathogen-associated molecular pattern, B-cell receptor, antigenic stimulation, cytokine stimulation, T-cell independent immune response, diffuse large B-cell lymphoma

## Abstract

B-lymphocytes play major adaptive immune roles, producing antibody and driving T-cell responses. However, how immunometabolism networks support B-cell activation and differentiation in response to distinct receptor stimuli remains incompletely understood. To gain insights, we systematically investigated acute primary human B-cell transcriptional, translational and metabolomic responses to B-cell receptor (BCR), Toll-like receptor 9 (TLR9), CD40-ligand (CD40L), interleukin-4 (IL4) or combinations thereof. T-independent BCR/TLR9 co-stimulation, which drives malignant and autoimmune B-cell states, jointly induced PD-L1 plasma membrane expression, supported by NAD metabolism and oxidative phosphorylation. BCR/TLR9 also highly induced the transaminase BCAT1, which localized to lysosomal membranes to support branched chain amino acid synthesis and mTORC1 hyperactivation. BCAT1 inhibition blunted BCR/TLR9, but not CD40L/IL4-triggered B-cell proliferation, IL10 expression and BCR/TLR pathway-driven lymphoma xenograft outgrowth. These results provide a valuable resource, reveal receptor-mediated immunometabolism remodeling to support key B-cell phenotypes including PD-L1 checkpoint signaling, and identify BCAT1 as a novel B-cell therapeutic target.

## Introduction

B-cells decode a multitude of membrane receptor stimuli to decide whether and how to respond to myriad innate and adaptive immune stimuli. Collective B lymphocyte responses to receptor signals drive humoral and cell-mediated immune responses, but also underlie autoimmune and B-cell lymphoma disease states. In addition to their obligatory role in humoral immunity, B-cells also carry out major immune functions, including the initiation of T-cell responses, immune homeostasis and as a driver of tumor responses to checkpoint blockade^1, 2^. Yet, much remains to be learned about how human B-cell immunometabolism response to distinct T-cell dependent versus T-cell independent signals, received either alone or in combination, drive rapid immune responses.

B-cells recognize a remarkable range of antigens via the cell surface B-cell receptor (BCR), comprised of immunoglobulin heavy and light chains and associated CD79a/Iga and CD79b/Igb signaling chains. Following BCR activation, immunogens are internalized and processed in lysosomes, where peptide antigens are presented via major histocompatibility complex class II molecules to CD4+ T-cells. In turn, activated CD4+ T-cells can then provide crucial second signals to drive B-cell activation, and two signals are generally needed to drive B-cell proliferation^2^. These include CD40-ligand (CD40L/CD154) and interleukin-4 (IL4)^3^. CD40L trimers activate cognate B-cell plasma membrane CD40 receptors, which stimulate NF-kB, MAP kinase and AKT/PI3K pathways^4, 5^, whereas the IL4-receptor (IL4R) stimulates JAK/STAT pathways to drive B-cell activation and differentiation^6^. T-cell CD40L and cytokine cues are critical for major B-cell activities, including germinal center formation, class-switch recombination and somatic hypermutation. Receipt of multiple activating signals rescues B-cells from death^7^ and induces rapid B-cell proliferation, which serves to expand the pool of antigen-specific B-cells, supports B-cell differentiation and the formation of germinal centers.

B-cells can also be activated by innate immune signals, including pathogen associated molecular patterns (PAMP) that are recognized by Toll-like receptors (TLR). TLR9 recognizes unmethylated CpG dinucleotides within endosomal compartments, where it then signals through the adaptor protein MyD88 to activate interleukin-1 receptor associated kinases (IRAK) 1 and 4 to activate NF-kB, MAPK and interferon regulatory factor pathways^8–13^. PAMPs provide the adaptive immune system with an additional layer of self/non-self discrimination^14, 15^. TLR9 signaling, together with BCR stimulation, drives type 1 T-independent responses^2^. By contrast, certain highly multivalent antigens trigger type 2 T-independent responses^16^. However, TLR9 can downmodulate antigen presentation and disrupt affinity maturation downstream of BCR engagement^17^. TLR9/BCR co-activation drives the formation of the internalized MyD88-TLR9-BCR (My-T-BCR) complex, which hyperactivates mTOR from late endosomes^18^. It remains to be characterized how My-T-BCR intersects with nutrient sensing pathways that control mTOR activity. Gain-of-function CD79 and MyD88 mutations hyper-activate BCR/TLR9 signaling in several types of lymphoma, including the diffuse large B-cell lymphoma (DLBCL) MCD subtype^19–21^. MCD DLBCL are aggressive and typically have inferior clinical outcomes, highlighting the need for novel therapeutic approaches^20^. TLR signaling also plays key roles in B-cell autoimmune responses, including in systemic lupus erythematosus^8, 22, 23^. Interestingly, although TLR9 promotes loss of tolerance to DNA in lupus, it is protective against systemic lupus erythematosus through MyD88 independent roles^24, 25^.

An open question is how immunometabolism networks support B-cell activation, differentiation, rapid proliferation and humoral responses to distinct stimuli. Whereas resting primary human B-cells have low basal metabolism^26^, B-cells rapidly remodel metabolism pathways in response to receptor stimuli^7, 27, 28^. For example, human B-cells rapidly increase oxidative phosphorylation (OXPHOS) and glycolysis in response to BCR stimulation, but are unable to sustain this in the absence of T-cell help or TLR9 co-stimulation^7^. IL4 co-stimulates B-cell responses, in part through increasing the abundance of α-ketoglutarate (αKG), a key TCA intermediate and anaplerotic substrate^29^.

To gain insights into *ex vivo* primary human B-cell responses, we leveraged bulk transcriptomic, proteomic and metabolomic approaches to characterize responses to nine major routes of immune receptor stimulation. BCR/TLR9 jointly induced PD-L1 plasma membrane expression through effects at the transcriptional and post-translational levels in a manner dependent on NAD metabolism and oxidative phosphorylation. BCR/TLR9 co-activation also highly induced the enzyme BCAT1 and drove its lysosomal subcellular localization, where it synthesized branched chain amino acids to support mTOR hyper-activation critical for primary B-cell growth and survival. A BCAT1 antagonist diminished outgrowth of BCR/TLR9-driven lymphomas *in vivo*, including a patient derived xenograft.

## Results

### Multi-omic profiling of differential primary human B-cell responses to immune receptor stimuli

**Figure 1:**
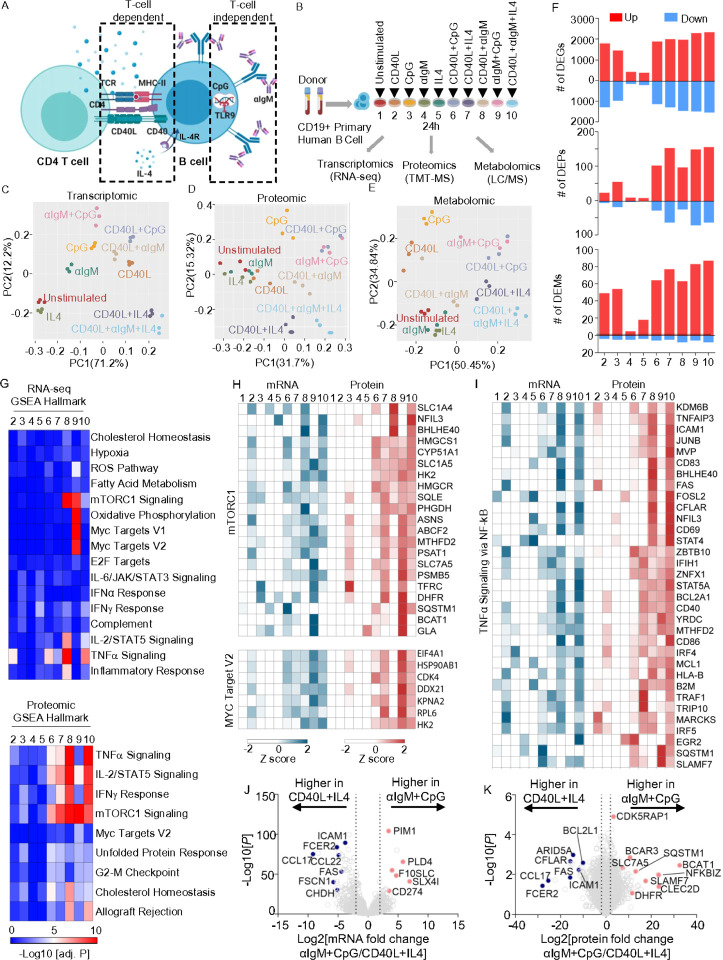
Receptor-driven B-cell activation results in different cellular responses. A) Schematic of T-cell dependent or independent B-cell activation pathways. B) Multi-omic profiling experimental design. Human primary peripheral blood CD19+ B-cells were isolated by negative selection from n=3 donors and stimulated by CD40L (50ng/ml), CpG (0.5μM), IL4 (20ng/ml), αIgM (1mg/ml) or combinations 24 hours and then profiled. C-E) Principle component analysis of transcriptomic (C), proteomic (D) and metabolomic (E) datasets. F) Numbers of differentially expressed genes (DEGs), proteins (DEP) and metabolites (DEMs) across conditions using the numbering scheme in (B), relative to unstimulated cells and using a p-value < 0.01 and a fold change > 2 or <0.5 cutoff. G) Gene Set Enrichment Analysis (GSEA) of pathways enriched across conditions at the RNA (top) or protein (bottom) levels. H) Heatmap visualization of row Z scores of mRNA and protein abundances of GSEA Hallmark mTORC1 signaling (top) and MYC target V2 (bottom) geneset. I) Heatmap visualization of row Z scores of mRNA and protein abundances of GSEA TNFa signaling via NF-kB geneset. J) Volcano plot visualization of -Log10 (p-value statistical significance) vs Log2 (mRNA abundance foldchange) from RNAseq analysis of αIgM+CpG vs CD40L+IL4 stimulated B-cells. K) Volcano plot visualization of -Log10 (p-value statistical significance) vs Log2 (protein abundance foldchange) from proteomic analysis of αIgM+CpG vs CD40L+IL4 stimulated B-cells.

To systematically investigate acute primary human B-cell transcriptional, translational and metabolomic responses to key T-cell dependent vs T-cell independent receptor cues, peripheral blood CD19+ cells were purified by negative selection. We modeled responses to key T cell independent stimuli by anti-immunoglobulin cross-linking to drive BCR signaling and/or with the TLR9 activating PAMP CpG oligonucleotide. To model responses to key T-dependent stimuli, B-cells were instead stimulated with trimeric CD40 ligand (CD40L) and/or interleukin-4 (IL4) (Fig. 1A–B). We also activated B-cells with CD40L + CpG to model bystander B-cell activation or with CD40L + aIgM ± IL4 to model antigen receptor stimulation with T-cell help. We profiled cells at 24 hours post stimulation, a timepoint prior to the first mitotic division, in order to facilitate cross-comparison with the basal unstimulated state. Indicative of distinct biological outcomes, B-cells exhibited markedly different morphological in response to these stimuli. For instance, B-cells stimulated by αIgM+CpG appeared larger but formed smaller aggregates by comparison with cells stimulated by T-dependent signals (Extended data Fig. S1A).

In order to systematically investigate how individual vs combinatorial stimuli altered B-cell expression and immunometabolism networks, we conducted parallel RNA-based sequencing (RNA-seq), tandem-mass-tag mass spectrometry proteomic and polar metabolite liquid chromatography/mass spectrometry (LC/MS) profiling across all 10 conditions (Supplemental Table S1). Principle component analysis (PCA) analysis yielded triplicates that closely clustered together from each condition, indicating a high level of reproducibility across conditions and human donors (Fig. 1C–E). Notably, transcriptomic responses to T-dependent stimuli modeled by CD40L + IL4 ± aIgM clustered oppositely from responses to T-independent αIgM+CpG (Fig. 1C).

We next cross-compared differentially expressed genes (DEGs), differentially expressed proteins (DEPs) and differentially expressed metabolites (DEMs) across the 10 conditions to broadly characterize stimulus-specific B-cells responses. Response magnitude was generally higher at the transcriptomic than proteomic level at this early timepoint, with CD40L or CpG stimulation eliciting larger numbers of DEGs than stimulation by either aIgM or IL4 alone (Fig. 1F, Extended Data 1B). Interestingly, similar numbers of DEGs were observed in response to CD40L, CpG and to the combinatorial stimuli tested, suggesting dominant effects of these ligands. Combinatorial stimulation differentially regulated a large gene set, with 1,077 DEGs overlapping across all five combinatorial stimulation groups, despite these varying by the degree of T-dependent vs T-independent signaling (Extended Data Fig. 1C).

Despite a degree of overlap, we nonetheless observed that each of the 9 stimulation conditions most dynamically regulated a small set of genes often implicated in B-cell biology, most of which were upregulated (Extended Data Fig. 2). These included well-characterized T cell-dependent genes upregulated by CD40/IL4, including *AICDA*, which encodes the enzyme AID, and XBP1, which stimulates plasma cell differentiation^30^. Interestingly, IL4 stimulation alone most highly induced the transcriptional repressor BCL6, which is critical for germinal center formation and for preventing premature B-cell activation and differentiation^31, 32^ (Extended Data Fig. 2). By contrast, T-cell-independent αIgM + CpG co-stimulation selectively induced a wider range of targets, including the *de novo* nicotinamide adenine dinucleotide biosynthetic enzyme NAMPT and the neutral amino acid transporter SLC7A5 (Extended Data Fig. 2). CpG alone, but to a greater extent αIgM + CpG highly induced *PRDM1*, which encodes BLIMP1, the master regulator of antibody secreting cell differentiation. Gene Set Enrichment Analysis (GSEA)^33^ highlighted that αIgM + CpG stimuli more strongly induced mTORC1 signaling, MYC targets, and OXPHOS (Group 9, Fig. 1G–H), suggesting that T-independent stimulation may preferentially upregulate these key immunometabolic pathways. By contrast, CD40L+IL4 or CD40L+IL4+αIgM stimulation more strongly induced TNFα, IL-2/STAT5, and Interferon γ signaling, (Group 7, Fig. 1G and I). These gene sets included transcription factors, metabolic enzymes, and cell surface receptors with critical roles in B-cell adhesion, activation, survival and antigen presentation (Fig. 1H), highlighting multiple-levels of T/B-cell crosstalk.

To gain further insights into B-cell responses to T-dependent vs T-independent stimuli, we next directly cross-compared the CD40L+IL4 versus αIgM+CpG conditions. Volcano plot analysis highlighted CD40L+IL4 induction of mRNAs encoding the T-cell chemoattractant chemokines CCL17 and CCL22, exemplifying B/T cells cross-communication even at the early 24 hour timepoint. Transcripts encoding multiple cell B-cell surface proteins were likewise more highly induced by T-dependent signaling, including FAS, ICAM-1 and CD23/FCER2. By contrast, αIgM+CpG more highly upregulated CD274 which encodes the immune checkpoint regulator PD-L1 (Fig. 1J). On the protein level, CD40L+IL4 more strongly upregulated the anti-apoptotic proteins CFLAR/cFLIP and BCL2L1/Bcl-xL, even at this early timepoint, whereas αIgM+CpG more highly induced CLEC2D, the lectin receptor for the natural killer inhibitor receptor KLRB1. Taken together with effects on PD-L1, this result suggests that T-independent stimulation may down-modulate key cellular immune responses. Suggestive also of key T-independent effects on B-cell immunometabolism, αIgM+CpG also more highly induced dihydrofolate reductase (DHFR), the neutral amino acid transporter SLC7A5 and branched chain amino acid transaminase 1 (BCAT1), which are key regulators of folate, amino acid and branched chain amino acid metabolism, respectively (Fig. 1J–K).

### BCR and TLR9 co-activation hyperactivates key B-cell immunometabolism pathways

**Figure 2:**
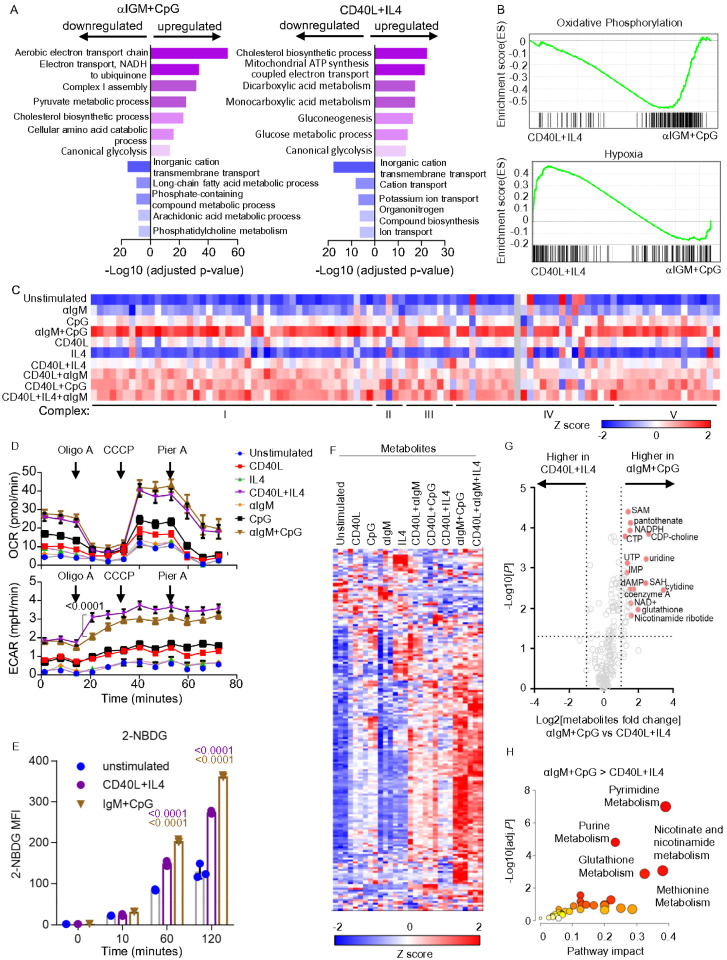
Cross-comparison of αIgM+CpG versus CD40/IL4 driven B-cell metabolism remodeling. A) Gene Ontology (GO) Biological Process analysis of genes differentially expressed in αIgM+CpG vs CD40L+IL4 stimulated B-cells, using a curated metabolism gene set^34^. B) GSEA Hallmark pathway analysis of oxidative phosphorylation (top) and hypoxia) genes in CD differentially expressed genes in αIgM+CpG vs CD40L+IL4 stimulated B-cells C) Heatmap analysis of mRNA encoding electron transport chain (ETC) components in cells stimulated as indicated. Columns display Z-score values for each ETC gene, produced by cross-comparison of the 10 conditions. D) Seahorse oxygen consumption rate (OCR, top) and extracellular acidification rate (ECAR, bottom) of primary-B-cells stimulated by indicated conditions for 24h and subject to flux analysis in the presence of the indicated ETC inhibitors. Mean ± SEM from n = 7 replicates are shown. P values were calculated by 2-way ANOVA. E) FACS analysis of primary B-cell glucose analogue 2-NBDG uptake at the indicated time points post-stimulation. Mean ± SEM from n=3 replicates. P values were calculated by Student t test. F) Heatmap analysis showing intracellular metabolites Z-scores in primary human B-cells stimulated for 24h as indicated. G) Volcano plot visualization of -Log10 (p-value statistical significance) vs Log2 (fold-change metabolite abundance) from primary B-cells stimulated by αIgM+CpG vs CD40L+IL4 for 24h, from n=3 replicates. H) MetaboAnalyst Pathway enrichment analysis of metabolites that were higher in αIgM+CpG than CD40L+IL4 stimulated cells at 24h.

To gain further insights into how T-dependent vs independent stimuli impact primary B-cell immunometabolism, we next analyzed metabolic gene responses, using a curated gene set^34^. αIgM+CpG and CD40/IL4 similarly induced multiple metabolic pathways, albeit to varying degrees, indicating responses to somewhat overlapping metabolic demands (Fig. 2A). Yet, large clusters of metabolic genes were more highly induced by αIgM+CpG than by CD40L + IL4 (Extended Data Fig. **3A**). Gene ontology (GO) analysis indicated that OXPHOS, fatty acid metabolism, purine metabolism and amino acid catabolism were all more highly induced by αIgM+CpG than by CD40L + IL4 (Fig. 2A–B, Extended Data **3A-B**).

Components of all five electron transport chain (ETC) complexes were the most highly upregulated by αIgM+CpG, whereas stimulation by CD40L-containing regimen induced these to a lesser extent (Fig. 2C). Phenotypically, Seahorse metabolic flux analysis identified that αIgM+CpG and CD40L+IL4 nonetheless similarly induced basal respiration and maximal respiratory capacity, perhaps suggesting post-transcriptional level compensatory regulation (Fig. 2D). Consistent with prior analyses^7^, combinatorial αIgM+CpG stimuli more strongly induced oxygen consumption rate (OCR) than either αIgM or CpG alone. A similar phenomenon was observed with CD40L and IL4 stimuli (Fig. 2D). Of the transcription factors implicated in control of respiratory chain component expression, MYC was the most highly induced by CD40L and regimen containing CD40L, although combinatorial αIgM + CpG also highly induced MYC (Extended Data Fig. 3C). Notably, CD40 + IL4 co-stimulation elicited higher extracellular acidification rate (ECAR), a measure of glycolysis (Fig. 2D). Taken together with our GSEA analysis, which identified hypoxia gene upregulation in CD40L/IL4 stimulated cells (Fig. 2B), these results suggest that T-dependent B-cell responses may be more reliant on aerobic glycolysis. However, while CD40L/IL4 and αIgM/CpG stimulation each significantly increased glucose uptake, αIgM+CpG did so more strongly (Fig. 2E).

Across individual stimuli, CD40L or CpG more highly impacted the intracellular B-cell metabolite landscape than either αIgM or IL4, consistent with the magnitude of their transcription level effects. αIgM+CpG produced the strongest cellular metabolome-wide effect (Fig. 2F), which triggered higher metabolite levels of the purine and pyrimidine nucleotide, methionine, nicotinate/nicotinamide and glutathione metabolism pathways (Fig. 2G–H, **Supplementary Table S1**). Despite these differences, combinatorial CD40L+IL4+αIgM stimulation, which models a key T-dependent GC light zone B-cell stimulus, produced somewhat overlapping metabolomic responses with T-independent αIgM+CpG stimulation (Fig. 2F and Extended Data Fig. **3A-B**). Collectively, these findings highlight that distinct types of receptor stimuli, including T-dependent vs independent regimen, produce myriad metabolomic responses in primary human CD19+ B-cells, with potential major effects on key humoral immune phenotypes.

### BCR and TLR9 activation upregulates plasma membrane PD-L1 expression

**Figure 3:**
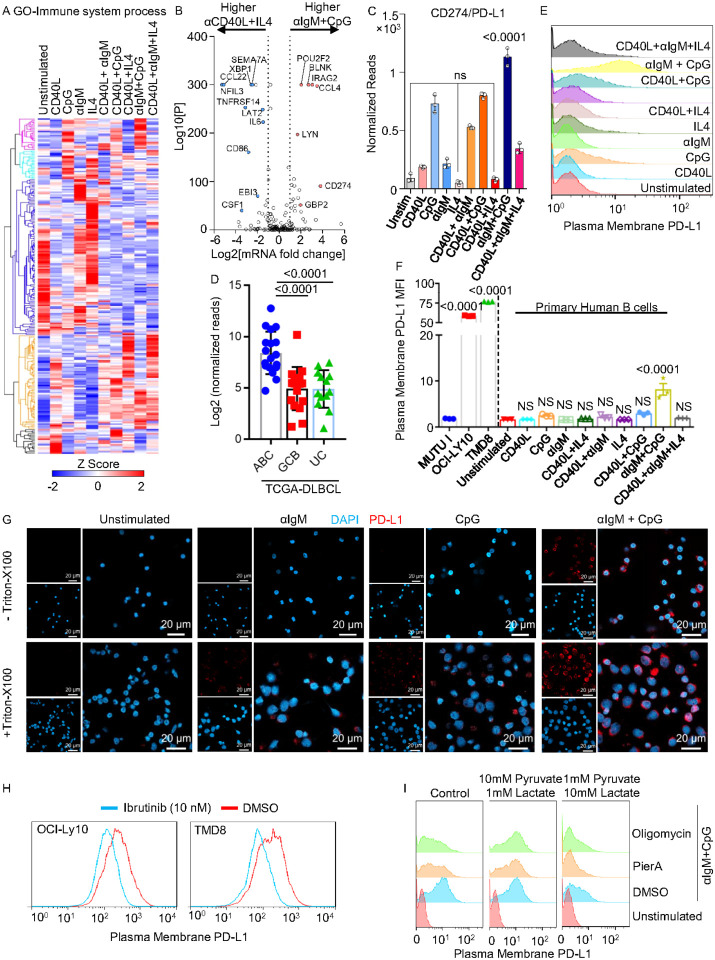
BCR/TLR9 co-stimulation jointly induces PD-L1 expression and plasma membrane trafficking. A) Gene Ontology Immune System Process gene mRNA Z-score hierarchical clustering analysis in primary human B-cells stimulated for 24h, as indicated. B) Volcano plot visualization of -Log10 (p-value statistical significance) vs Log2 (fold-change mRNA abundance) from RNAseq of αIgM+CpG vs CD40L+IL4 stimulated primary B-cells.GO Immune system process genes are displayed. C) Mean ± SEM normalized PD-L1 RNAseq reads from primary B-cells stimulated as indicated for 24h. P values were calculated by multiple t tests using Holm-Sidak method. D) Mean ± SEM Log2 normalized PD-L1 RNAseq from TCGA cohort^81^ DLBCL tumors. ABC, activated B-cell DLBCL; GCB, germinal center B-cell DLBCL; UC, uncharacterized. P values were calculated by Student t test. E) FACS analysis of plasma membrane PD-L1 expression in primary human B-cell stimulated as indicated for 24h. F) Mean ± SEM normalized median fluorescence intensity (MFI) of plasma membrane PD-L1 expression in primary human B-cells stimulated for 24h, as in (E), or in Burkitt lymphoma Mutu I or MCD DLBCL OCI-LY10 and TMD8 cells with CD79B/MYD88 gain-of-function mutations from n=3 replicates. P values were calculated by Student t test. G) Confocal microscopy analysis of PD-L1 (red) expression in non-permeabilized (- Triton-X100) or permeabilized (+ Triton-X100) primary B-cells stimulated for 24 hours as indicated. Scale bar, 20mM. H) FACS analysis of plasma membrane PD-L1 expression in OCI-LY10 or TMD8 incubated with DMSO or ibrutinib for 48h. Representative of n=2 replicates. I) FACS analysis of plasma membrane PD-L1 expression in primary-B-cells stimulated by aIgm/CpG for 24h, as indicated, and also treated with DMSO, 100nM piericidin A (PierA) or 10nM oligomycinA for 1 hour before stimulation and then cultured in unsuplemented media or media with 10mM pyruvate/1mM lactate or 1mM pyruvate/10mM lactate. Representative of n=3 replicates.

To further characterize B-cell responses to distinct receptor stimuli, we next used the GO Immune System Process gene set to perform heatmap analysis. This analysis highlighted distinct clusters of immune genes that preferentially responded to each condition (Fig. 3A). Volcano plot cross-comparison of the αIgM+CpG vs. CD40L+IL4 Immune System Process genes further revealed genes differentially regulated by T-dependent vs independent stimulation. For instance, multiple CD40L + IL4 induced transcripts are encoded by known NF-kB targeted genes and drive pro-inflammatory and T-cell responses, including the cytokines IL-6, CSF-1 and EBI3, the chemokine CCL22 and the T-cell costimulatory ligand CD86/B7–2 (Fig. 3B). By contrast, CD274/PD-L1 was 14-fold more highly upregulated by αIgM + CpG, suggesting opposing effects on T-cell responses. Interestingly, the BCR signaling mediators BLNK and LYN and the octamer binding transcription factor POU2F2/OCT-2 were amongst the most highly αIgM+CpG differentially upregulated genes (Fig. 3B), suggesting a positive-feedback loop.

We next cross-compared PD-L1 mRNA abundances across all 10 conditions. Whereas CpG upregulated PD-L1 expression, alone or even more highly together with aIgM, we noticed the opposite effect for IL4: cells stimulated by CD40 or CD40L/aIgM had significantly higher PD-L1 levels than those stimulated by CD40/IL4 or CD40L/aIgM/IL4, respectively (Fig. 3C). Consistent with this result, PD-L1 levels were higher in two activated B-cell (ABC) DLBCL, a subset of which have driver mutations that activate BCR and MyD88 signaling, than in tumors of the germinal center B-cell subtype, which tend to have lower levels of BCR and TLR signaling (Fig. 3D). FACS analysis confirmed that plasma membrane PD-L1 expression was high on the MCD DLBCLs OCI-LY10 and TMD8, but low on a Burkitt lymphoma cell line that lacks activated BCR or TLR signaling (Fig. 3E).

Despite robust PD-L1 mRNA induction, we noticed that TLR9 stimulation alone resulted in only modest plasma membrane PD-L1 expression (Fig. 3E–F). To explore whether concurrent BCR signaling was therefore important for PD-L1 translation or trafficking, we stained PD-L1 in stimulated primary human B-cells, with or without Triton-X100 permeabilization. Consistent with our FACS analysis, only intracellular PD-L1 was evident in αIgM or CpG stimulated cells. By contrast, combined αIgM+CpG stimulation resulted not only in higher total PD-L1 levels, but also in PD-L1 plasma membrane expression (Fig. 3G). Likewise, the Bruton’s tyrosine kinase antagonist ibrutinib, which blocks a key aspect of BCR signaling, decreased PD-L1 plasma membrane expression in OCI-LY10 and TMD8 (Fig. 3H).

Since BCR/TLR9 co-stimulation highly induced OXPHOS gene expression (Fig. 2A–C), we next tested effects of the ETC complex I inhibitor piericidin A or the ATP synthase inhibitor oligomycin A on PD-L1 expression. Interestingly, each impaired plasma membrane PD-L1 induction by αIgM+CpG (Fig. 3I). Consistent with a key reductive stress role in this phenotype, PD-L1 expression could be rescued in either Piericidin A or oligomycin treated cells by αIgM+CpG stimulation in the presence of 10mM pyruvate, whose conversion to lactate regenerates NAD from NADH. By contrast, addition of 10 mM lactate prevented αIgM+CpG driven PD-L1 induction even in DMSO treated primary B-cells (Fig. 3I). Since the oncometabolite R-2HG negatively impacts complex V activity, we next tested its effects on αIgM+CpG mediated PD-L1 induction. Interestingly, R-2HG, but not its isomer alpha-ketoglutarate (aKG), significantly reduced PM PD-L1 expression (Extended Data Fig. 4A-B). Furthermore, R-2HG reduced PD-L1, but not CD19 expression in a dose-dependent manner in OCI-LY10 cells with hyperactive BCR/TLR9 signaling (Extended Data Fig. 4C-D). Taken together, these results suggest a key role for αIgM + CpG-driven redox homeostasis in cell surface PD-L1 expression and suggest a key advantage for MCD DLBCL cells in co-activating these pathways.

### BCAT1 is essential for BCR/TLR9 driven B-cell proliferation.

**Figure 4:**
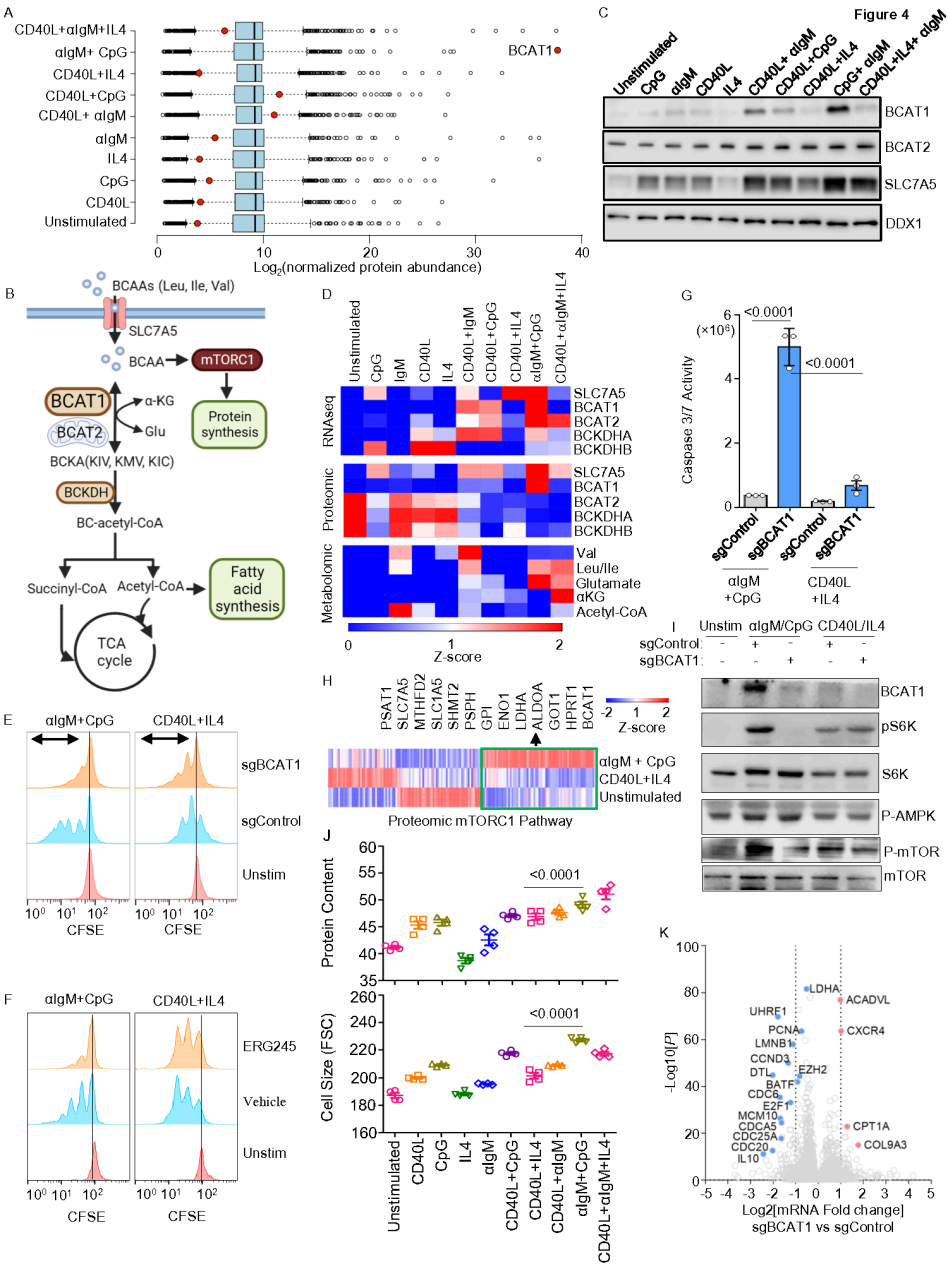
BCR/TLR9 co-stimulation highly induces BCAT1, which is essential for aIgM/CpG but not CD40/IL4-driven primary B-cell mTORC1 activation, growth and survival. See also Fig. S5. A) Boxplot of Log2 normalized protein abundances from proteomic analysis of primary B-cells stimulated for 24h, as indicated. BCAT1 protein levels are highlighted by red circles. B) Branched chain amino acid (BCAA) metabolism schematic. Cytosolic BCAT1 and mitochondrial BCAT2 catalyze the reversible transamination of branched chain keto acids (BCKA) and BCAA. C) Immunoblot of BCAT1, BCAT2, SLC7A5 and load control DDX1 from whole cell lysates (WCL) of B-cells stimulated for 24h, as indicated. D) Heatmap analysis of key BCAA pathway RNA, protein and metabolite Z-scores in primary B-cells stimulated as indicated for 24h. E) Carboxyfluorescein succinimidyl ester (CFSE) analysis of primary B-cells that were untreated or electroporated with Cas9 ribonucleoprotein complexes loaded with control (sgControl) or BCAT1 targeting sgRNA (sgBCAT1) and then stimulated for 5 days, as indicated. Representative of n = 3 replicates. F) CFSE analysis of primary B-cells treated with vehicle or BCAT1 inhibitor ERG-245 (100 mM) and stimulated as indicated. Representative of n = 3 replicates. G) Mean ± caspase 3/7 activity of primary-B-cells electroporated with the indicated Cas9 RNPs and then stimulated as indicated for 48 hours. P values were calculated by Student t test. H) Heatmap analysis of selected mTORC1 pathway target gene Z-scores, shown in vertical colums, in primary B-cells stimulated for 24h as shown. Selected metabolic enzymes upregulated by αIgM + CpG, but not by CD40L+IL4 stimulation are highlighted. I) Immunoblots of WCL from primary-B-cells expressing the indicated Cas9 RNPs and stimulated as indicated for 24h. Representative of n = 3 replicates. J) FACS analysis of total protein content (top) and forward side scatter (FSC) cell size (bottom) in primary B-cells stimulated for 24h as indicated and labeled with eBioscience efluor 670 (5μM). MFI ± SEM from biological quadruplicate replicates are shown. K) Volcano plot visualization of -Log10 (p-value statistical significance) vs Log2 (fold-change mRNA abundance) from RNAseq of primary B-cells electroporated with Cas9 RNPs loaded with control or BCAT1 sgRNAs and stimulated by αIgM+CpG for 24h, from n=3 datasets. P values were calculated by multiple t tests using Holm-Sidak method.

We observed that BCR/TLR9 co-stimulation markedly upregulated Branched Chain Amino Acid Transaminase 1 (BCAT1) expression on the mRNA and protein levels, whereas it was induced to a much lesser extent or not at all by the other stimuli (Fig. 4A, Extended Data 5A). BCAT1 is an aminotransferase that can either synthesize or catabolize the branched chain amino acids (BCAA) leucine, isoleucine and valine in reversible reactions, but has not previously been studied in B-cell activation. When running in the forward direction, BCAT1 converts the nitrogen donor glutamine and branched chain ketoacids (BCKA) into alpha-ketoglutarate (aKG) and BCAA, which support protein synthesis and mTOR activation. When running in the reverse direction, BCAT1 instead catabolizes aKG and BCAA to produce glutamine and BCKA, which fuel the tricarboxylic acid cycle (TCA) and fatty acid synthesis^35–37^ (Fig. 4B). We validated that αIgM + CpG more strongly up-regulated BCAT1 by immunoblot. By comparison, the mitochondrial BCAT2 isoform was expressed in unstimulated cells and was only modestly upregulated by any of the conditions (Fig. 4B–C). Consistent with a key BCAA role in support of BCR/TLR9 co-stimulated B-cells, the major plasma membrane BCAA transporter SLC7A5 was also highly induced (Fig. 4B–D). BCAA abundances were also higher in αIgM + CpG stimulated cells than in B-cells stimulated by combinatorial regimens with the exception of CD40L+aIgM+IL4. By contrast, subunits of the BCKA dehydrogenase complex (BCKDHA/B), which participate in BCKA catabolism to acetyl-CoA and CO_2_ were downmodulated on the protein level (Fig. 4D). This raises the interesting possibility that BCAT1 may take on a selectively important role downstream of CD79 and MyD88.

To gain insights into BCAT1 roles in B-cell activation, we tested the effects of BCAT1 perturbation on proliferation and survival of primary B-cells stimulated by αIgM+CpG versus by CD40L+IL4. We electroporated freshly isolated primary human B-cells with Cas9 ribonucleoprotein complexes containing control or *BCAT1* targeting single guide RNA (sgRNA) (Extended Data **Fig. 5B**)^38^. Intriguingly, CRISPR *BCAT1* knockout (KO) strongly impaired αIgM+CpG but not CD40L+IL4 driven primary B-cell outgrowth, as judged by carboxyflouorescein succinimidyl ester (CFSE) dye dilution assay (Fig. 4E, Extended Data 5C). Similar results were obtained with primary B-cells treated with the highly selective leucine-based BCAT1 small molecule antagonist ERG245^39^, suggestive of on target effects at the level of BCAT1 (Fig. 4F, Extended Data 5D-E). To then interrogate effects of BCAT1 KO on primary B-cell survival, we next performed caspase activity assays. BCAT1 KO induced caspase 3/7 activity to a significantly greater extent in αIgM+CpG than in CD40L+IL4 stimulated primary B-cells (Fig. 4G, Extended Data 5F).

We hypothesized that BCAT1 may be needed to support mTOR in αIgM+CpG stimulated cells, given that αIgM+CpG most highly activated GSEA Hallmark mTORC1 signaling at the RNA and protein levels (Fig. 1G). In support, proteomic analysis highlighted that clusters of mTORC1 pathway targets were more highly upregulated by αIgM+CpG than by CD40L/IL4 stimulation, including multiple components of the glycolysis, one-carbon metabolism and amino acid metabolism pathways (Fig. 4H). Consistent with this result, mTORC1 target S6K phosphorylation levels were higher in αIgM+CpG stimulated cells, and BCAT1 KO strongly impaired S6K phosphorylation (Fig. 4I). BCAT1 was similarly important for αIgM+CpG driven phosphorylation of mTORC1 serine 2448, which is indicative of mTOR activation. Additionally, immunoblot analysis revealed that αIgM+CpG induced a much higher level of phospho-S6, further indicating hyperactivated mTORC1 signaling. While phosphorylation of the mTOR negative regulator AMP kinase was somewhat higher in αIgM+CpG stimulated cells, this was not affected by BCAT1 KO, suggesting alternative route(s) by which BCAT1 supports mTOR. Since mTORC1 regulates translation, we also tested translation rate using puromycin pulse labeling, which detects puromycin incorporation into elongating protein chains^40^. Puromycin labeling indicated that αIgM+CpG more highly induced nascent polypeptide synthesis than the other conditions (Extended Data Fig. 5G). Similar results were observed by flow cytometry analysis of total protein content and also of cell size, which is also controlled by mTORC1^41^. However, total protein content was slightly higher in cells stimulated by CD40L+αIgM+IL4 (Fig. 4J). These findings indicate that BCAT1 is a major positive regulator mTOR in αIgM+CpG-stimulated B-cells.

To also gain insights into potential BCAT1 roles in transcription regulation downstream of CD79 and MyD88, we performed RNAseq on αIgM+CpG stimulated BCAT1 CRISPR KO versus control primary human B-cells. BCAT1 KO upregulated 145 and downregulated 101 B-cell genes. GO analysis indicated that BCAT1 depletion resulted in downregulation of E2F targets and G2-M checkpoint genes in αIgM+CpG stimulated cells (Extended Data Fig. 5H, **Supplemental Table S2**). The most highly downregulated genes included interleukin-10 (IL-10), the DNA methylation enzyme UHRF1, the transcription factor BATF, and the genes CDC25A, MCM10 and PCNA, each of which have key cell cycle roles (Fig. 4J). We validated that αIgM+CpG induced IL10 on the protein level in primary B-cells. Furthermore, BCAT1 KO reduced IL10 abundance to levels observed in CD40L/IL4 stimulated cells, in whom BCAT1 KO did not alter IL10 levels (Extended data Fig. 5I). Interestingly, BCAT1, IL-10 and PD-L1 mRNA abundances were each markedly higher at 48 hours of αIgM/CpG than CD40L/IL-4 stimulated cells (Extended Data Fig. 5J). Since IL10 is a B regulatory cell hallmark^42–46^, our data raise the possibility that BCAT1 may supports B regulatory cell function upon My-T-BCR activation.

### BCAT1 supports BCAA production in BCR/TLR9 stimulated B-cells

**Figure 5:**
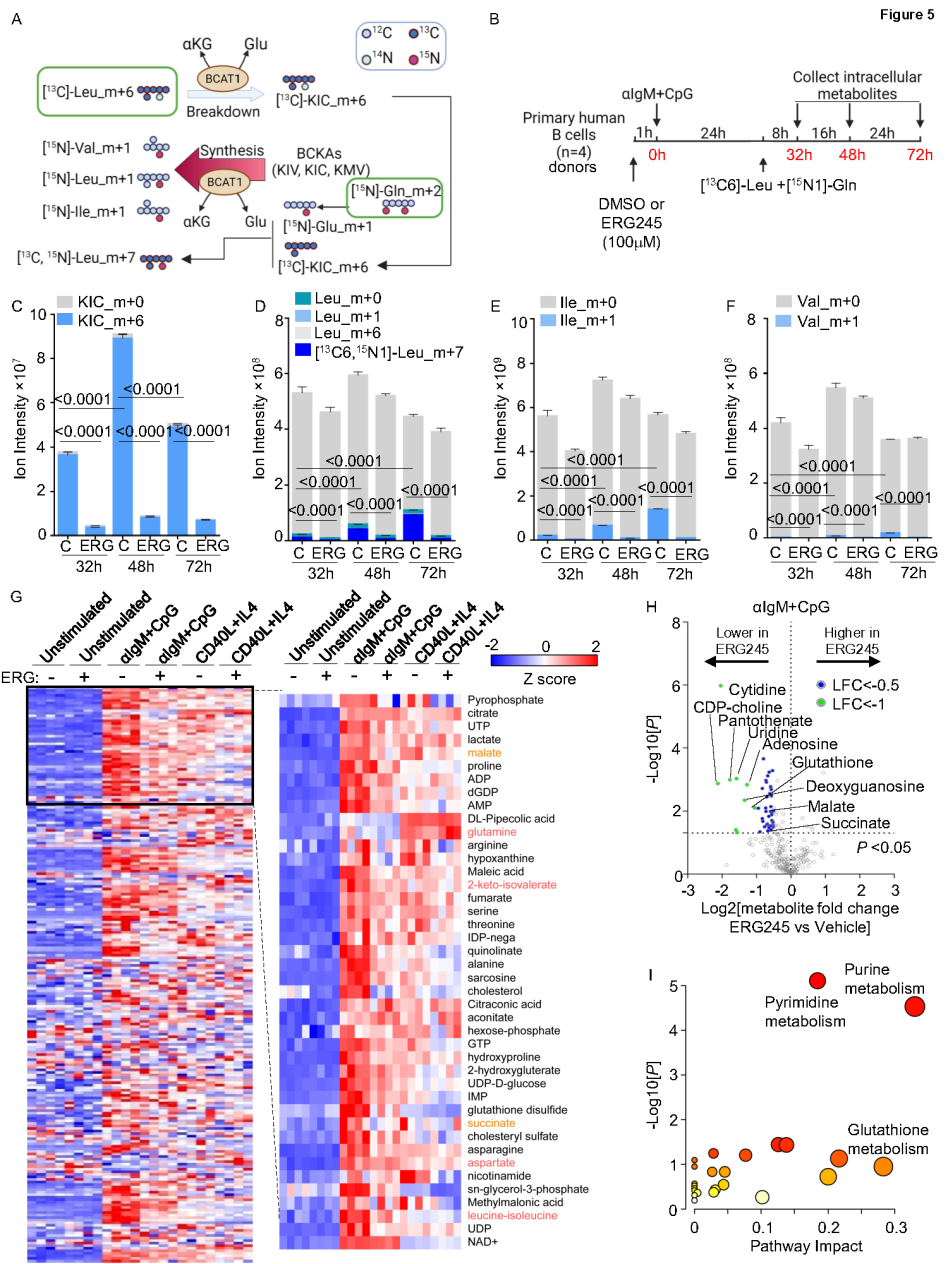
BCR/TLR9 Stimulation drives BCAA synthesis in human primary B-cells, related to Figure S6. A) Isotope tracing schematic. [^13^C]-L-Leucine_m+6 was used to trace BCAT1 BCAA catabolism to a-keto-isocaproic acid (KIC), while [^15^N]-Glutamine_m+2 was used to monitor BCAT1 BCAA biosynthesis from KIC, a-keto-isovaleric acid (KIV) or a-keto b-methylvaleric acid (KMV). Gln, glutamine. Glu, glutamate. B) Isotope tracing experimental design. Primary B-cells from n=4 donors were pre-treated with vehicle or ERG245(100μM) for 1h and then stimulated by αIgM+CpG for 24 hours in the presence of vehicle or ERG245. Cells were washed with PBS three times and resuspended in glutamine/leucine free media supplemented with 381mM ^13^C6-leucine and 2.054mM ^15^N2-glutamine + 10% dialyzed FBS. ERG245(100μM) and αIgM+CpG stimulants were also refreshed at this time point. Intracellular metabolites were profiled at 8, 24 and 48 hours later. C) Ion intensities of m+6 labeled and unlabeled KIC at the indicated times in cells treated with vehicle control (C) or ERG245. D) Ion intensities of labeled and unlabeled leucine (Leu) at the indicated times in cells treated with vehicle control (C) or ERG245. E) Ion intensities of labeled and unlabeled isoleucine (Ile) at the indicated times in cells treated with vehicle control (C) or ERG245. F) Ion intensities of labeled and unlabeled valine (Val) at the indicated times in cells treated with vehicle control (C) or ERG245. G) Heatmap analysis of metabolite Z-scores in primary B-cells treated with vehicle or ERG245 (100μM) and stimulated as indicated for 24h. H) Volcano plot visualization of -Log10 (p-value statistical significance) and Log2 (fold-change metabolites abundance) from metabolomic analysis of ERG245 vs vehicle treated primary B-cells stimulated by αIgM+CpG for 24h, from n=4 replicates. I) MetaboAnalyst Pathway enrichment analysis of metabolites diminished by ERG245 treatment in αIgM+CpG stimulated B cells at 24h.

We next used [^13^C]-leucine_m+6 and [^15^N]-glutamine_m+2 isotope tracing to investigate the directionality of BCR/TLR9 induced BCAT1 BCAA metabolism. To trace BCAT1 conversion of BCAA and αKG to BCKA and glutamate (Glu), we incubated αIgM+CpG stimulated cells with 0.381mM [^13^C]-leucine_m+6 to survey for the appearance of labeled BCKA catabolites. However, since BCKA can then be re-aminated by BCAT1 using glutamine (Gln) as the amino donor, we also added 2mM [^15^N]-Gln_m+2. We measured BCAA catabolism by detecting [^13^C]-ketoisocaproate (KIC)_m+6 levels. Likewise, we measured BCAA anabolism by detecting the appearances of [^15^N]-labeled leucine (Leu)_m+1, isoleucine (Ile) _m+1, and valine (Val) _m+1 (Fig. 5A). B-cells were pretreated with either vehicle or ERG245 for 1 hour before αIgM+CpG stimulation, then incubated in medium containing [^13^C]-Leu_m+6 and [^15^N]-Gln_m+2 from 24 hours post-stimulation. Labeled and unlabeled metabolite abundances were quantitated at 32-, 48-, and 72-hours post-stimulation (Fig. 5B).

Liquid chromatography/mass spectrometry metabolite tracing indicated that BCAT1 does contribute to B-cell BCKA pools upon its induction by αIgM+CpG, as [^13^C]-KIC_m+6 levels significantly increased between 32- and 72-hours post-stimulation. [^13^C]-KIC_m+6 levels then decreased at the 72 hour timepoint, potentially indicating a balance between production and consumption (Fig. 5C, **Supplemental Table S3**). BCAT1 inhibition by ERG245 strongly decreased [^13^C]-KIC_m+6 levels at all timepoints, indicating BCAT1 roles in KIC generation (Fig. 5C). However, we also observed steadily increasing [^15^N]-labeled Leu_m+1, Ile_m+1, and to a lesser extent Val_m+1 levels, each of which were suppressed by ERG245, indicating that BCAT1 also consumes glutamine to synthesize BCAA and produce aKG (Fig. 5D–F). [^13^C,^15^N]-Leu_m+7 comprised the majority of the [^15^N]-labeled Leu pool, indicating that BCAT1 preferentially reaminated [^13^C]-KIC_m+6 at this timepoint (Fig. 5D). However, the fraction of [^15^N]-labeled Val_m+1 was comparatively smaller, suggesting that BCAT1 may preferentially synthesize Leu and Ile at the early stage of αIgM+CpG-driven B-cell activation (Fig. 5D–F). In contrast to serving as a substrate for BCAA biosynthesis, [^13^C]-KIC_m+6 was not a major TCA cycle anaplerotic input. TCA intermediate m+2 isotope signals remained low throughout the timecourse, despite marked increases in unlabeled TCA metabolite abundances (Extended Data Fig. 6A-G). Intriguingly, ERG245 nonetheless strongly decreased levels of most TCA cycle intermediates, indicating that BCAT1 plays a crucial role in coordinating TCA metabolism in BCR/TLR9 co-activated cells, potentially via effects on mTOR (Extended Data Fig. 6A-G).

To broadly profile BCAT1 contributions to αIgM+CpG vs CD40L/IL4 co-activated primary B-cells, we performed LC/MS metabolome profiling on cells stimulated in the presence of DMSO vehicle control or ERG245. Whereas ERG245 had minimal effects on unstimulated cells, ERG245 broadly restrained BCR/TLR9 driven metabolite increases. Comparatively smaller effects were observed on CD40L/IL4 treated cells, and ERG245 only modestly affected the metabolome (Fig. 5G and **Supplemental Table S4**). Leucine-isoleucine and 2-keto-isovalerate levels were higher in αIgM+CpG stimulated cells, whereas glutamine was higher in CD40/IL4 stimulated cells (Fig. 5G and Extended Data 6H). Notably, ERG245 did not significantly change aspartate abundances, despite its being a substrate for similar transamination reactions (Extended Data Fig. 6I). Volcano plot and metabolism pathway impact analysis highlighted that ERG245 most strongly reduced abundances of nucleotides and glutathione in BCR/TLR9 stimulated B-cells, potentially due to effects at the level of mTOR (Fig. 5H–I) and reflecting that resting human B-cells have low nucleotide and glutathione levels and must rapidly increase them upon activation^26^. Together, these findings suggest that BCAT1 supports BCAA pools and mTOR upon BCR/TLR9-driven B-cell activation.

### BCR/TLR9 signaling targets BCAT1 to lysosome membranes to support mTOR

**Figure 6:**
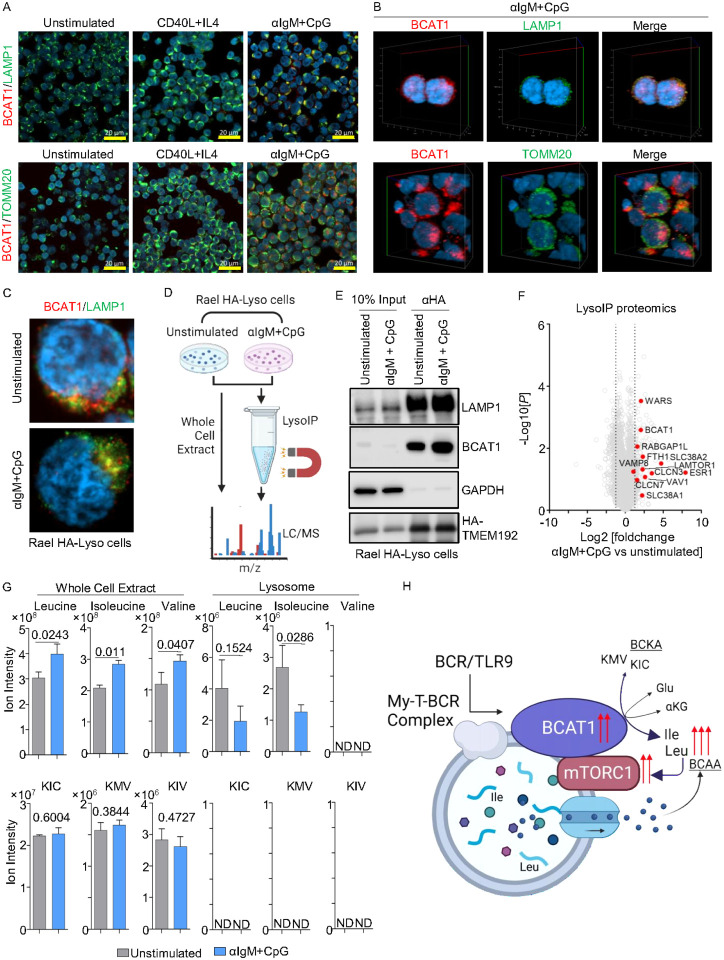
BCR/TLR9 but not CD40/IL4 co-stimulation targets BCAT1 to remodeled lysosomes, related to Figure S7. A) Confocal microscopy analysis of BCAT1 (red) co-localization with the lysosomal LAMP1 (top, green) or mitochondrial TOMM20 (bottom, green) markers in primary B-cells stimulated for 24h as indicated. B) 3D Z stack reconstruction of BCAT1, LAMP1 (top) or TOMM20 (bottom) in primary B-cells stimulated by αIgM+CpG for 24h. C) Confocal analysis of BCAT1 and LAMP1 co-localization in Rael TMEM192-HA+ B-cells (HA-Lyso cells) stimulated by αIgM+CpG for 24h, as indicated. D) Lyso-IP proteomic analysis workflow. E) Immunoblot of WCL or anti-HA immunopurified lysosomes from Rael Lyso cells stimulated as in (D). F) Volcano plot of -Log10 (p-value) vs Log2 (fold-change) of tandem-mass-tag protein abundances in immunopurified lysosomes from Rael Lyso-IP cells as in (D). G) Normalized BCAAs and BCKA ion intensities in whole cell vs lysosomes immunopurified from Rael Lyso-IP cells as in (D). H) Schematic of BCAT1 lysosomal targeting and BCAA production to support mTORC1 hyperactivation.

A complex of lysosomal membrane proteins sense amino acid levels to control mTORC1 recruitment and activation. When amino acids are abundant, mTORC1 is recruited to the outer lysosomal membrane, where it is activated by RHEB^47–50^. When leucine levels are low, several mechanisms block mTORC1 activation. Sestrin2 inhibits mTORC1 lysosomal recruitment and activation^51^, SAR1B inhibits the mTORC1 activator GATOR2^52^, leucyl-tRNA synthetase LARS fails to activate Rag GTPase^53^, and Raptor acetylation decreases to further downmodulate mTORC1^54^. As BCAT1 is thought to be cytoplasmic, we hypothesized that BCR/TLR9 induced BCAT1 supports mTORC1 by producing BCAA in close proximity with lysosomes. To test this, we performed confocal microscopy on primary B-cells at rest or 24 hours following stimulation by CD40L/IL4 versus aIgM + CpG, which revealed a high degree of colocalization between BCAT1 and lysosomal-associated membrane protein 1 (LAMP1), but not with the mitochondrial marker translocase of outer mitochondrial membrane 20 homolog (TOMM20) (Fig. 6A–B, Extended Data 7A-B). Thus, a major population of BCAT1 homes to lysosomes in BCR/TLR9 stimulated primary human B-cells, presumably to the lysosomal outer membrane as BCAT1 does not appear to contain a targeting sequence for lysosomal uptake.

To further investigate BCAT1 subcellular localization, we then leveraged the LysoIP approach, in which stably expressed HA-epitope tagged transmembrane protein 192 (TMEM192) is used as a bait for lysosomal affinity purification^55^ (Fig. 6C–D). We stably expressed TMEM192 in Rael Burkitt lymphoma Rael B-cells, in which BCAT1 and LAMP1 co-localization was increased by aIgM+CpG stimulation (Extended Data Fig. 7C). The lysosomal marker LAMP1 was enriched in material anti-HA-TMEM192 immunopurified from Rael cells, whereas cytosolic GAPDH was highly depleted, suggesting successful lysosome isolation. Importantly, BCAT1 was also enriched in immunopurified lysosomes, particularly following aIgM+CpG stimulation, even though in Rael cells total BCAT1 levels remained similar (Fig. 6E). Confocal microscopy again highlighted that aIgM+CpG increased BCAT1/LAMP1 co-localization in Rael TMEM192+ cells (Extended Data Fig. 7C). These results suggest that BCR/TLR9 signaling contributes to BCAT1 lysosomal subcellular localization, likely at the outer membrane.

To gain further insights into how BCR/TLR9 stimulation remodels Rael lysosomes, we performed LC/MS proteomic profiling of lysosomes immunopurified from aIgM+CpG versus unstimulated LysoIP Rael cells. Consistent with our immunoblot and microscopy analyses, BCAT1 was enriched in lysosomes purified from stimulated Rael cells (Fig. 6F, Supplemental Table S5). Intriguingly, BCR/TLR9 signaling also increased lysosomal levels of LAMTOR1, which has a key role in assembly of the Ragulator complex that together with the Rag GTPases control mTORC1 lysosomal recruitment^56, 57^. Further suggestive of cross-talk between My-T-BCR and mTORC1 at the level of the lysosome, SLC38A1/2 abundance was also increased in lysosomes of stimulated Rael cells. SLC38A1/2 are membrane transporters that specialize in the uptake of neutral amino acids and that are implicated in mTORC1 regulation in T-cells^58^, but have not yet been studied in B-cells (Fig. 6F, Supplemental Table S5). Of note, SLC38A2 family member SLC38A9 is a lysosomal membrane protein that is a major regulator of lysosomal amino acid sensing^59, 60^.

To then directly investigate BCR/TLR co-activation effects on lysosomal BCAA and BCKA, we performed targeted LC/MS analysis in whole cells, or in lysosomes immunopurified from resting versus αIgM+CpG stimulated Rael HA-LysoIP cells. Whole cell Leu, Ile and Val BCAA pools each significantly increased upon BCR/TLR9 co-stimulation. However, lysosomal Leu and Ile levels instead substantially decreased, suggesting that these amino acids were exported from lysosomes upon αIgM+CpG stimulation. Whole cell BCKAs remained unchanged by BCR/TLR stimulation, whereas BCKAs were not detected in immunopurified lysosomes (Fig. 6G). Collectively, our results support a model where BCAT1 augments Leu and Ile synthesis at the lysosomal membrane to support mTORC1 hyper-activation (Fig. 6H).

### BCAT1 inhibition suppressed MCD DLBCL tumor growth *in vitro* and *in vivo*

**Figure 7:**
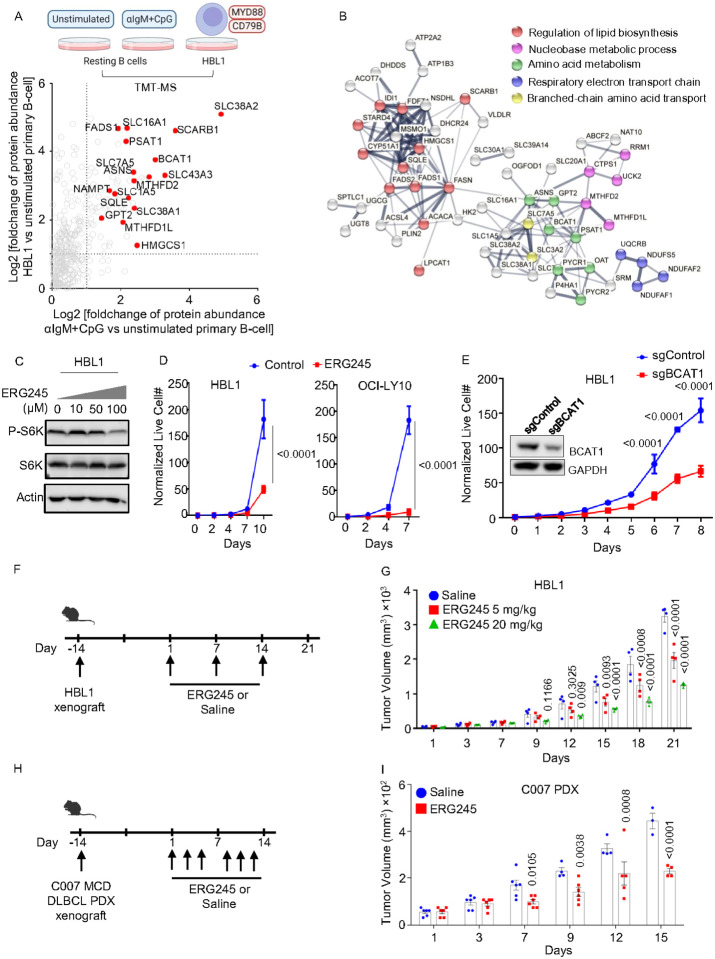
BCAT1 is highly expressed in BCR/TLR9 driven MCD DLBCL, where it supports B-cell proliferation in vitro and *in vivo*, related to Figure S7. A) Volcano plot comparing tandem-mass-tag proteomic log2(foldchange) of whole cell protein abundances in HBL1 DLBCL vs unstimulated primary B-cell (y-axis) vs 24h αIgM+CpG stimulated vs unstimulated primary B cell (x-axis). Proteins from a curated metabolic gene set are shown^34^. From n=3 proteomics dataset. B) String analysis of protein-protein interactions amongst factors upregulated in both αIgM+CpG stimulated and HBL1 (fold change >2) relative to unstimulated primary B-cells. C) Growth curve analysis of vehicle or 100μM ERG245 treated HBL1 (left) or OCI-LY10 MCD DLBCL cells. Mean ± SD values from n=3 replicates. D) Immunoblots of WCL from HBL1 cells treating with the vehicle, 10, 50, and 100μM ERG-245 as indicated for 24h. E) Growth curve analysis of Cas9+ HBL1 cells expressing control or BCAT1 sgRNAs. Mean ± SD values from n=3 replicates. F) Schematic of HBL1 MCD DLBCL mouse xenograft experiments. HBL1 tumors were implanted in mouse flanks two weeks prior to administration of vehicle vs 5mg/kg or 20mg/kg ERG245. G) Mean ± SEM HBL1 tumor volumes in mice treated as indicated. H) Schematic of C007 MCD DLBCL patient derived xenograft (PDX) experiments. Tumors were implanted in mouse flanks two weeks prior to administration of vehicle vs 20mg/kg ERG245. I) Mean ± SEM C007 PDX tumor volumes in mice treated as indicated.

mTORC1 is hyper-activated by the My-T-BCR complex in MCD DLBCL, where BCR, TLR9 and MyD88 form a super-complex that co-localizes with mTORC1 on endolysosomes^18^. To gain insights into whether TLR9/BCR co-activation causes similar remodeling in primary B-cells and in MCD DLBCL, we cross-compared proteomes from aIgM/CpG-stimulated primary B-cells or the tumor-derived MCD DLBCL HBL1 cell line with resting primary B-cells. Interestingly, a group of metabolic proteins were similarly upregulated in αIgM+CpG and in HBL1, presumably by My-T-BCR signaling in both contexts, including BCAT1 and SLC7A5. Using a fold change of ≥2 as the threshold, we identified that 1280 proteins were commonly upregulated by αIgM+CpG and in HBL1, relative to their resting primary B-cell levels (Fig. 7A, **Supplemental Table S6**). STRING analysis^61^ identified multiple metabolic subnetworks upregulated in both activated B-cell contexts, including branched-chain amino acid metabolism (Fig. 7B).

To determine if BCAT1 activity is likewise important for MCD DLBCL proliferation in vitro, we tested ERG245 effects on HBL1 and OCI-LY10. ERG-245 reduced phospho-S6 levels, indicating its inhibitory effects on mTOR signaling (Fig. 7C). ERG245 significantly reduced proliferation of both MCD cell lines (Fig. 7D), which was further supported by the observation that CRISPR editing of BCAT1 also impaired HBL1 proliferation (Fig. 7E). To extend this observation *in vivo,* we established HBL1 xenografts in NOD.Cg-Prkdcscid Il2rgtm1Wjl/SzJ (NSG) mice. HBL1 tumors were grown for approximately 14 days post-implantation, until tumor volumes reached 32–64 mm^3^. Mice were then treated weekly with either vehicle control or ERG245 at doses of 5 or 20 mg/kg, via intraperitoneal injection (Fig. 7F). There was no significant difference in body weight observed between mice treated with vehicle control versus ERG245 at either the 5 or 20 mg/kg dose over the following three weeks (Extended data Fig. 7E), indicating that ERG245 was well-tolerated. However, tumor volumes were significantly smaller from day 12 onwards in ERG245 treated mice, in a dose-dependent manner (Fig. 7G). Tumor volumes of OCI-LY10 xenografts were also significantly reduced by ERG245 (Extended data Fig. 7F). To further extend these results, we next tested ERG245 effects on a MCD DLBCL patient derived xenograft (PDX) model. 2 weeks following establishment of the C007 PDX, ERG245 was dosed 3 times per week at 20 mg/kg (Fig. 7H). ERG245 significantly decreased PDX tumor volumes, beginning at day 7 post ERG245 dosing, and also significantly increased body weight of C007 PDX carrying mice (Fig. 7I, Extended data Fig. 7G).

## Discussion

B lymphocytes are uniquely positioned to integrate a wide range of antigenic, PAMP and T cell cues^1, 2, 62–65^. As a result, it is hypothesized that a ‘signaling code’ drives distinct B-cell responses to receptor stimuli28. However, much has remained to be learned about how key T-cell dependent versus independent B-cell stimuli remodel immunometabolism networks to control B-cell activity. Here, we present a multi-omic compendium of acute primary human CD19+ peripheral blood B-cell responses to BCR, TLR9, CD40 and/or IL-4R activation, each of which are prominent drivers of naive B-cell responses. We found that BCR and TLR9 jointly induce PD-L1 as well as the transaminase BCAT1, which is targeted to lysosomes to support mTORC1 hyper-activation.

CpG alone strongly upregulated primary human B-cell PD-L1 mRNA, in agreement with a recent study^66^. Unexpectedly, BCR co-activation was also required to induce PD-L1 plasma membrane expression. OXPHOS and reductive stress further modulated PD-L1 expression. Consistent with the finding that NAD supports PD-L1 expression^67^, BCR/TLR9 co-stimulation hyper-induced NAMPT. However, in contrast to this prior study which found a key NAD-dependent interferon regulatory factor 1 (IRF1) role in PD-L1 induction, BCR/TLR9 did not induce IRF1, suggesting a distinct mechanism of NAD-regulated PD-L1 induction. It will therefore be of interest to explore whether the mitochondrial complex I inhibitor metformin synergizes with checkpoint blockade. B-cells are abundant in the tumor microenvironment (TME), where they play major roles in therapeutic responses to checkpoint therapy^68, 69^. B-cell rich tertiary lymphoid structures are observed in subtypes of sarcoma, melanoma and renal cell carcinoma that have improved responses to checkpoint blockade^70–72^. Our results raise the possibility that BCR/TLR signaling, perhaps in response to tumor antigens and nucleic acids released by dying tumor cells in the necrotic core, may support B-cell checkpoint signaling in the TME to drive PD-L1 expression.

Immunometabolic regulation is critical for supporting B-cell proliferation and effector functions^28, 73^. Our data suggests that BCR/TLR9 induce BCAA production by BCAT1 at the lysosome membrane to support mTORC1 hyperactivation, B-cell growth and survival. How BCAT1 homes to lysosomal membranes remains an intriguing question. Since this has not been observed in other cell types, an intriguing possibility is that My-T-BCR itself may recruit BCAT1 to endolysosomes^18^. Alternatively, BCR/TLR9 stimulation causes major lysosomal remodeling and may induce a protein or post-translational modification to target BCAT1 to lysosomes. In support, highly spatially delineated roles are emerging as a theme in BCAT biology. For instance, a distinct spatially constrained mitotic spindle BCAT1-localized role was recently observed in epithelial cells^74^, and BCAT2 also exerts a spatially regulated role, in which it forms a mitochondrial BCAA metabolon together with branched-chain α-keto acid dehydrogenase to shuttle BCAA catabolites into the TCA cycle^75^.

While BCAT1 catalyzes reversible transamination reactions, BCAA catabolism typically predominates^76, 77^. We provide evidence that BCAT1 fluxes in both directions in BCR/TLR9-stimulated cells, but that the majority of labeled leucine was [^13^C,^15^N]-leucine (m+7), suggesting that BCAA synthesis predominates. We speculate that the specific location of BCAT1 can determine its catabolic and anabolic activity. To test this hypothesis, subcellular mass spectrometry imaging^78^ could be used to measure [^13^C,^15^N]-leucine in the lysosomal vicinity. Also suggestive of an anabolic role, BCR/TLR9 did not increase protein levels of the branched-chain a-keto acid dehydrogenase complex (BCKDC), which catalyzes the irreversible conversion of BCKA to acetyl- and succinyl-CoA for TCA metabolism. Notably, BCAT1 promotes BCAA production in BCR-ABL driven chronic myelogenous leukemia, in which BCAT1 blockade impairs B-cell proliferation and causes differentiation^77^.

BCAT1 is expressed in CD4+ T-cells, where it drives BCAA catabolism to instead downmodulate mTORC1 activity^79^. BCAT1 also catabolizes BCAA in activated macrophages^36^. Interestingly, CD8 T-cell BCAT1 instead supports effector functions, though does not influence BCAA levels^39^. We speculate that differences in glutamine and BCKA levels may account for these differences. Notably, increased glutamate levels in EZH2-mutant acute myelogenous leukemia drive BCAA production by BCAT1 to support mTORC1 and also to restrict aKG levels^80^. It will therefore be of interest to determine whether BCAT1 homes to lysosomes in these other hematopoietic cell contexts.

Novel therapeutic targets are needed for the treatment of a wide range of pathological B-cell states^64^. BCAT1 may therefore constitute an intriguing metabolic vulnerability, including in MCD DLBCL and in certain autoimmunity states, including systemic lupus erythematosus, where BCR/TLR7 drives pathology. Since BCAT1 inhibition also increases CD4+ T-cell mTORC1 activation and ameliorates CD8+ T-cell exhaustion, BCAT1 antagonists may be particularly promising for DLBCL. BCAT1 antagonists may also exert synergy with glutaminase to further reduce BCAA levels and mTORC1. BCAT1 may also serve as a novel biomarker for pathological BCR/TLR9-driven B-cell states.

In summary, we used multi-omic profiling to systematically characterize primary human B-cell responses to key receptor stimuli. Collectively, our studies provide a major resource for primary human B-cell immunometabolism investigation. We identified major immunometabolism pathways that differ on the transcriptional, proteomic and metabolomic levels with receptor-driven metabolism reprogramming. BCR/TLR9, but not CD40/IL4 co-stimulation, highly induced BCAT1, which trafficked to lysosomal membranes to support BCAA synthesis and mTORC1 hyperactivation. BCAT1 was critical for BCR/TLR9 but not CD40/IL4 driven primary B cell growth and survival. BCAT1 inhibition significantly impaired growth of BCR/TLR9-pathway dependent MDC DLBCL xenografts *in vivo*, identifying BCAT1 as a promising novel B-cell lymphoma therapeutic target.

## Methods

### Cell lines and reagents

Rael Burkitt cells, HBL1, OCI-LY10 and TMD8 DLBCL cells were obtained from Ethel Cesarman. HBL1 with stable *Streptococcus pyogenes* Cas9 expression were generated by lentiviral transduction and blasticidin selection (5 μg/ml), as previously described^82^. Rael with stable TMEM192-HA were generated by lentiviral transduction using pLJC5-Tmem192–3xHA (Addgene plasmid #102930, a gift from David Sabatini). B-cells were grown in RPMI 1640 medium (Gibco, Life Technologies) with 10% fetal bovine serum (FBS, Gibco) in a humidified incubator at 37°C with 5% CO2 and routinely certified as mycoplasma-free, using the MycoAlert kit (Lonza). 293T were obtained from ATCC and grown in Dulbecco’s Modified Eagle’s Medium (DMEM) with 10% FBS. For selection of transduced cells, puromycin was added at the concentration of 3 μg/ml. The BCAT1 inhibitor ERG245 was obtained from Adonia E. Papathanassiu and used at 100 μM for *in vitro* experiments. ERG-24 was used at 5mg/kg or 20mg/kg for *in vivo* mouse xenograft experiments, as indicated. Sodium pyruvate and lactate (Sigma-Aldrich) were used at 1:9 or at 9:1 mM ratios. Oligomycin was used at 10nM. Piericidin A was used at 100nM. Ibrutinib was used at 0.01μM. R-2-hydroxyglutarate (R-2HG) and alpha-ketoglutarate (αKG) were used at 100μM. All cell lines were routinely tested for mycoplasma by the Lonza Mycoalert kit, according to the manufacturer’s instructions. Antibodies used in the study was listed in Key Resources table.

### Primary Human B-cells.

Discarded, de-identified leukocyte fractions left over from platelet donations were obtained from the Brigham and Women’s Hospital Blood Bank or from the Gulf Coast Medical Center following collection of informed consent. Blood cells were collected from platelet donors following institutional guidelines. Since these were de-identified samples, the gender was unknown. Our studies on primary human blood cells were approved by the Brigham & Women’s Hospital Institutional Review Board. B-cells from Gulf Coast Medical Center were used for RNAseq analyses. Primary human B-cells were isolated by negative selection using RosetteSep Human B-cell Enrichment and EasySep Human B-cell enrichment kits (Stem Cell Technologies), according to the manufacturers’ protocols. B-cell purity was confirmed by FACS analysis of plasma membrane CD19 positivity. Cells were then cultured with RPMI 1640 with 10% FCS. 7×10^6 B-cells were used for each stimulation condition. Stimulants were added at the following concentrations: CD40L 50ng/ml (Enzo Life Sciences), CpG 0.5μM (IDT), IL4 (R&D systems) 20ng/ml and aIgM at 1ug/ml. Cells were treated in cultured in the absence of stimulation or stimulated by, CD40L only, CpG only, IL4 only, aIgM only, CD40L+CpG, CD40L+IL4, CpG+ aIgM, CD40L+ aIgM, and CD40L+ aIgM+IL4. Cells were treated for 24 hours. For each experiment, cells from 3 donors were isolated and treated separately. At 24 hours, cells were counted and viability was measured using Trypan Blue staining and counted on a TC20 automated cell counter (Bio-Rad).

### B-cell line CRISPR-Cas9 editing

CRISPR/Cas9 engineering was performed using stable Cas9 expression and Broad Institute Brunello library sgRNA sequences. sgRNA oligos were obtained from Integrated DNA Technologies and cloned into the pLentiGuide-Puro vector (Addgene plasmid #52963, a gift from Feng Zhang). Lentiviruses were produced in 293T cells by co-transfection of pLentiGuid-puro with psPAX2 and VSV-G packaging vectors. At 24 hours post transfection, the cell culture media was changed to RPMI-1640+10% FBS. Two rounds of lentiviral transduction were performed at 48 and 72 hours post-transfection. Transduced cells were selected by puromycin (3 μg/ml), added 48 hr post-transduction. Depletion of target gene encoded protein expression was confirmed by immunoblot.

### Primary B-cell CRISPR-Cas9 editing using crRNA-tracrRNA-Caspase 9 RNP system

To create a RNP complex for knocking out BCAT1 in human primary-B-cells, a mixture of 2.2 μL crRNA, 2.2 μL tracrRNA, and 5.6 μL duplex buffer was heated to 95°C for 5 minutes and then cooled to room temperature. The crRNA-tracrRNA complex was then combined with 0.6 μL of caspase 9 to assemble the RNP complex, which was incubated for 20 minutes at room temperature. The RNP complex was mixed with washed human primary-B-cells suspended in buffer T, then electroporated using parameters of 1700V, 20ms width, and 1 pulse. Finally, the cells were added to prewarmed media. To enhance the knock-out efficiency, a 1:1 mixture of two BCAT1 crRNA-tracrRNA-Cas9 RNP complexes was electroporated simultaneously.

### Immunoblot analysis

Immunoblot was performed as previously described^83^. In brief, whole cell lysates (WCL) prepared by boiling cells in 1× Laemmli buffer were separated by SDS-PAGE electrophoresis, transferred onto the nitrocellulose membranes, blocked with 1% BSA in TBST buffer and then probed with relevant primary antibodies at 4 °C overnight, followed by secondary antibody incubation for 1 h at room temperature. Blots were then developed by incubation with ECL chemiluminescence for 1 min (Millipore) and images were captured by Licor Fc platform. Bands intensities were measured where indicated by Image Studio Lite Version 5.2. All antibodies used in this study were listed in the Key Resources Table.

#### Puromycin analysis of protein translation.

Two million cells were seeded at 0.3 million per ml in RPMI-1640+10% FBS. Puromycin (10 mg/ml) was added for 20 min at 37°C. WCLs were prepared and analyzed by immunoblot, using an anti-puromycin monoclonal antibody to visualize newly synthesized polypeptides.

### Flow cytometry analysis

Flow cytometry was performed on a BD FacsCalibur instrument. For live cell antibody staining, 1× 10^6^ cells were washed twice with FACS buffer (PBS, 1mM EDTA, and 0.5% BSA) and then stained with fluorophore conjugated PD-L1 or IL-10 primary antibodies for 30 minutes on ice. Labeled cells were then washed three times with FACS buffer prior to the flow cytometry. For CFSE staining, primary B-cells were stained with 10 μM CFSE for 15 minutes at 37°C, washed, resuspended at 100,000 cells/mL and then treated with indicated conditions. 2NBDG (2-(N-(7-nitrobenz-2-oxa-1,3-diazol-4-yl)amino)-2-deoxyglucose; ThermoFisher) was used to assess the real-time glucose uptake of human primary-B-cells by flow cytometry, as described previously^26^. eBioscience^™^ Cell Proliferation Dye eFluor^™^ 670 (5uM) was used for measuring total protein content in human primary-B-cells following the manuals. Cells were then analyzed by FACS. FACS data were analyzed with FlowJo V10.

### Growth curve analysis and caspase activation assay

Cells were counted and then normalized to the same starting concentration, using the CellTiterGlo (CTG) luciferase assay (Promega, Cat#G7570). Live cell numbers were quantitated at each timepoint by CTG measurements, and values were corrected for tissue culture passage. Fold change of live cell number at each timepoint was calculated as a ratio of the value divided by the input value. For long-term assays, chemicals were refreshed at every 72 hours in fresh media. Caspase 3/7 activity was quantified by Caspase-Glo assays (Promega) according to manufacturer’s manual, and normalized to the cell number of the same sample determined by CTG assay. All values were quantitated on a Molecular Devices plate reader.

### RNAseq analysis

Total RNA was isolated by the RNeasy Mini kit (Qiagen), following the manufacturer’s manual. An in-column DNA digestion step was included to remove the residual genomic DNA contamination. To construct indexed libraries, 1 μg of total RNA was used for polyA mRNA-selection, using the NEBNext Poly(A) mRNA Magnetic Isolation Module (New England Biolabs), followed by library construction via the NEBNext Ultra RNA Library Prep Kit (New England Biolabs). Each experimental treatment was performed in triplicate. Libraries were multi-indexed, pooled and sequenced on an Illumina NextSeq 500 sequencer using single-end 75 bp reads (Illunima) at the Dana Farber Molecular Biology core. Adaptor-trimmed Illumina reads for each individual library were mapped back to the human GRCh37.83 transcriptome assembly or EBV Akata genome (assession#: KC207813.1) using STAR2.5.2b^84^. Feature Counts was used to estimate the number of reads mapped to each contig^85^. Only transcripts with at least 5 cumulative mapping counts were used in this analysis. DESeq2 was used to evaluate differential expression (DE)^86^. DESeq2 uses a negative binomial distribution to account for overdispersion in transcriptome datasets. It uses a conservative analysis that relies on a heuristic approach. Each DE analysis used pairwise comparison between the experimental and control groups. Differentially expressed genes were identified and a p values < 0.05 and absolute fold change > 2 cutoff was used. Differentially expressed genes were subjected to Enrichr analysis which was employed to perform gene list-based gene set enrichment analysis on the selected gene subset. The algorithm used to calculate combined scores was described previously^87^. P value and log2 fold change were generated with DESeq2 under default settings with Wald test and normal shrinkage, respectively. Top 5 Enrichr terms that passed the adjusted p-value cutoff were visualized using Graphpad Prism 7.

Volcano plots were built with Graphpad Prism7. Heatmaps were generated by feeding Z-score values of selected EBV genes from DESeq2 into Morpheus software (https://software.broadinstitute.org/morpheus/).

### Confocal microscopy

Cells were seeded on glass slides in PBS, air dried and then fixed with 4% paraformaldehyde (PFA) in PBS for 10 minutes. PFA was removed and fixed cells were permeabilized with 0.1% Triton-X in PBS. Slides were blocked with 1% IgG-free BSA (Sigma-Aldrich, Cat# A2058) in PBS for 30 minutes at room temperature. Cells were incubated with primary antibodies against BCAT1 (Cell Signaling, 1:200) and LAMP1 (Santa Cruz, 1:100) or TOMM20 (Santa Cruz, 1:100) in PBS containing 1% BSA for 1 hour at 37°C. Slides were then washed three times and then incubated with secondary antibodies (Alexa Fluor 488-conjugated goat anti-mouse and Alex Fluor 594-conjugated goat anti-rabbit, diluted 1:250 in PBS) for 1 hour at 37°C. Slides were washed three times in PBS and incubated with 100 uL of Hoechst 33258 (10 μg/mL in PBS) for 10 minutes. Cells were then washed three times with PBS. ProLong Gold anti-fade was applied to the slide, which was then sealed with a No. 1.5 coverslip. Image acquisition was performed with the Zeiss LSM 800 instrument. Image analysis was performed with the Zeiss ZEN Blue software.

### Lysosomal immunopurification (LysoIP)

20 million cells were collected for each LysoIP. The cells were rinsed with PBS twice, then centrifuged for 2 minutes at 1000 g at 4°C, resuspended in 1 mL KPBS (136 mM KCl, 10 mM KH2PO4, pH 7.25 and was adjusted with KOH).The cells were homogenized with 55 strokes of a 2 mL homogenizer. The homogenized mixture was centrifuged for 2 minutes at 1000 g at 4°C. Anti-HA magnetic beads were prewashed with KPBS. 50 uL of the beads were placed in a 1.5 mL tube, mixed with 175 uL KPBS, and then separated using a magnetic stand. The supernatant was incubated with 25 uL of the KPBS prewashed anti-HA magnetic beads for 15–30 minutes (3 minutes for metabolite extraction). Immunoprecipitated lysosmes were washed 3 times with 300 uL KPBS using a DynaMag Spin Magnet. All buffers used in the experiment were pre-chilled on ice. For immunoblot analysis 100 uL of SDS-PAGE sample buffer was added to the tube and mixed with vortexing and boiled for 10min. For proteomics analysis, proteins from the isolated lysosomes were extracted by 5% formic acid. For targeted metabolomics, lysosomal metabolites were extracted with dry-ice cold 80% methanol.

### Intracellular metabolite profiling

For profiling for 10 stimulation conditions of human primary-B-cells, 5×10^6^ cells were seeded into a T25 flask with 10 mL of fresh RPMi-1640, supplemented with 10% FBS. 24 hours after seeding, 3×10^6^ cells were pelleted and resuspended in fresh media for additional 3 hours prior to intracellular metabolite extraction.

For profiling of ERG245 treated human primary-B-cells, 5×10^6^ cells were treated with vehicle or 100μM ERG245 1 hour prior to αIgM+CpG or CD40L+IL4 stimulation. At 24h post stimulation, 3×10^6^ live cells from each condition were pelleted and resuspended in fresh RPMI-1640 media, supplemented with 10% dialyzed FBS for 3 hours prior to intracellular metabolite extraction.

Cells were pelleted and washed with 5 mL of room temperature PBS. Pellets were resuspended in 1 mL of dry ice cold 80% methanol, incubated at −80°C for 30 minutes and centrifuged at 21,000 × g for 5 minutes to precipitate proteins. For mice xenograft tumors, 100mg of tumor tissues were washed once with PBC and submerged in 500 μl of 80% (vol/vol) HPLC-grade methanol (cooled to −80 °C). The tissues were smashed/grinded for 1–2 min with small pestle/tissue grinder on dry ice in the tube, vortex for 1 min at 4–8 °C and incubate for 4 hours at −80 °C. The tissues were then centrifuged at 21,000g for 10 min using a refrigerated centrifuge (4–8 °C). The supernatant was collected in pre-chilled tubes and stored at −80°C. On the day of analysis, supernatants were incubated on ice for 20 minutes and clarified by centrifugation at 21,000 × g at 4°C. At the Beth Israel Mass Spectrometry core, supernatants were dried down in a speed vacuum concentrator (Savant SPD 1010, Thermofisher Scientific) and resuspended in 100μL of 60/40 acetonitrile/water. The samples were then vortexed, sonicated in ice-cold water for 1 minute, and incubated on ice for 20 minutes. Supernatants were collected in an autosampler vial after centrifugation at 21,000 × g for 20 minutes at 4°C. Pooled QC samples were generated by combining ~15μL of each sample. Metabolite profiling was performed using Dionex Ultimate 3000 UHPLC system coupled to Q-Exactive plus orbitrap mass spectrometer (ThermoFisher Scientific, Waltham, MA) with an Ion Max source and HESI II probe operating in switch polarity mode. A zwitterionic Sequent zic philic column (150 × 2.1mm, 5μm polymer, part # 150460, MilliporeSigma, Burlington, MA) was used for polar metabolite separation. Mobile phase A (MPA) was 20mM ammonium carbonate in water, pH9.6 (adjusted with ammonium hydroxide) and MPB was acetonitrile. The column was held at 27°C, injection volume 5μL, autosampler temperature 4°C and LC conditions at flow rate of 0.15 mL/min were: 0min: 80% B, 0.5min: 80% B, 20.5min: 20% B, 21.3min: 20%B, 21.5min: 80% B with 7.5min of column equilibration time. MS parameters were: sheath gas flow 30, aux gas flow 7, sweep gas flow 2, spray voltage 2.80kV for negative & 3.80kV for positive, capillary temperature 310°C, S-lens RF level 50 and aux gas heater temp 370°C. Data acquisition was done using Xcalibur 4.1 (ThermoFisher Scientific) and performed in full scan mode with a range of 70–1000m/z, resolution 70,000, AGC target 1e6 and maximum injection time of 80ms. Data analysis was performed in Compound Discoverer 3.1 and Tracefinder 4.1. Samples were injected in randomized order and pooled QC samples were injected regularly throughout the analytical batch. Metabolite annotation was done base on accurate mass (±5ppm) and matching retention time (±0.5min) as well as MS/MS fragmentation pattern from the pooled QC samples against in-house retention time +MSMS library of reference chemical standards. Metabolites with CV<30% in pooled QC were used for the statistical analysis. The quality of integration for each metabolite peak was reviewed. Metabolites with p-values < 0.05, log_2_(fold change)>1 or <−1 were used for pathway analysis using MetaboAnalyst 5.0 (https://www.metaboanalyst.ca/MetaboAnalyst/ModuleView.xhtml).

### BCAT1 Isotope Tracing

B-cells were treated with ERG245 for 1 hour prior to aIgM+CpG stimulation. 24hr later, cells were incubated with 2 mM of ^15^2N -Glutamine and 381 μM of ^13^C_6_-leucine for 8, 16 and 24 hours. Equal number of cells were spun down for 5 minutes at room temperature and the supernatant was aspirated off. Metabolism was quenched by addition of 1 mL of dry ice cold methanol. Samples were vortexed upon methanol addition. Samples were stored at −80°C until analysis. On the day of analysis, samples were thawed on ice and then centrifuged at 21,000g and 4°C for 20 minutes. Supernatants were dried down using the Genevac EZ-2 elite vacuum dryer. Dried extracts were re-suspended in 120μL of water, vortexed, sonicated for 2 minutes in an ice-cold water bath, and clarified by spinning down at 21,00g for 20 minutes at 4°C. 5 μL of supernatant was injected onto a HSS-T3 column (2.1×100 mm, 1.8μM, part # 186003539 Waters Corporation). Column oven and autosampler temperatures were 30°C and 4°C respectively. The mobile phase A (MPA) was 0.1% formic acid in water and mobile phase B (MPB) was 0.1% formic acid in methanol. LC gradient conditions at a flow rate of 0.3mL/minute were: 0 min: 2.5% B, 1.5 min: 2.5%B, 8 min- 65%B, 9– 90% B, 10.8 min: 90% B, 11min : 2.5% B and 15 min : 2.5 % B. Metabolite detection was performed using a Dionex Ultimate 3000 UHPLC system coupled to a Q-Exactive Plus orbitrap mass spectrometer (ThermoFisher Scientific, Waltham, MA) with an Ion Max source and HESI II probe operating in polarity switching mode. MS parameters were: sheath gas flow = 30, aux gas flow = 7, sweep gas flow = 2, spray voltage = 2.80 for negative & 3.30 for positive ion modes, capillary temperature = 310°C, S-lens RF level=50, aux gas heater temp: 370°C. Data acquisition was performed using an Xcalibur 4.1 (ThermoFisher Scientific) in full scan mode with a range of 70–1000m/z, a resolving power of 70,000, an AGC target of 1 × 10^6^ and maximum injection time of 100 ms. Data analysis was done using Tracefinder4.1 software. Natural abundance correction was performed using IsoCorrectoR (Heinrich et al., 2018)

### Proteomics LC-MS/MS analysis

Sample Preparation for Mass Spectrometry Experiments (10 conditions)

Samples were prepared for LC-MS/MS experiments are described previously1. Clarified flash-frozen lysates were thawed and reduced by the addition of 5mM tris(2-carboxyethyl)-phosphine (TCEP) and reactive cysteines were blocked by alkylation through the addition of iodoacetamide (IAA) to a final concentration of 10mM. To prevent overalkylation, the reaction was quenched by the addition dithiothreitol (DTT) to a concentration of 15mM. A trigger channel was created by mixing 10uL from each lysate. Samples were than precipitated by the chloroform-methanol method.

Precipitated treated lysates were digested overnight with Lys-C (Wako), carried out at room temperature, at an approximated 1:100 protease:protein. This was followed by the addition of sequencing grade Trypsin (Promega) at 37°C for 6 hours for further proteolytic digestion. Digested samples were labelled at room temperature with TMT-11 reagents for 90 minutes and then quenched with hydroxylamine. To check labelling efficiency and overall relative sample abundance, 2μL from each TMT-labelled sample were combined to create a ratio check as samples were normalized by cell count. Samples were then combined with equal volume following the assumption of equal cell counts except for the anti-IgM+CpG sample. Review of the ratio check indicated that the sample treated with anti-Igm+CpG was over 4x more abundant than the least abundant sample. As such, the amount of anti-IgM+CpG sample added to the final combination was halved to reduce the effects of compositional proteomics2.

The combined TMT-labelled sample was then flash-frozen and dried down by vacuum centrifugation before resuspension in a formic acid solution such that pH of sample was below 3 (approximate final concentration of formic acid was 3%). The acidified sample was then desalted through a C18 solid phase extraction column (Waters) and dried down by vacuum centrifugation. Samples were resuspended in a 10mM ammonium bicarbonate and 5% acetonitrile solution for off-line basic pH reversed phase (BPRP) fractionation.

Sample Preparation for Mass Spectrometry Experiments (3 conditions)

Samples were prepared for LC-MS/MS analysis as described above except samples were normalized to total protein amount. A protein bicinchoninic acid assay was used on clarified lysate before reduction and alkylation. Additionally, after labelling samples with TMT-11 reagents, samples were normalized based on ratio check data to ensure a 1:1:1:1:1:1:1:1:1 total protein ratio was observed as described previously1.

### Off-line BPRP Fractionation

TMT-labelled samples suspended in 10mM ammonium bicarbonate and 5% acetonitrile were subjected to off-line BPRP high performance liquid chromatography on an Agilent 1100 pump with degaser and photodiode array detector. Elution occurred over a 13–37% acetonitrile 50-minute gradient in 10mM ammonium bicarbonate. The instrument separated the TMT-labelled sample into 96 fractions, collected in plate for by an automated fraction collector. To reduce redundancy, the 96 fractions were combined into 24 fractions as described previously. These 24 fractions were dried down by vacuum centrifugation and resuspended in 1% formic acid. A final cleanup step was performed by desalting each fraction over C18 stop-and-go extraction tips (STAGE tips), before resuspension in a loading buffer comprised of 5% acetonitrile and 5% formic acid.

### LC-MS/MS Experiments

For each BPRP fractionated sample, 12 of the 24 fractions were selected for LC-MS/MS analysis to reduce redundant identifications. Each fraction was analyzed on a Thermo Orbitrap Fusion Lumos with a Proxeon EASY-nLC 1200 system before the source (Thermo Fisher Scientific, San Jose). The mass spectrometer was operated in a data-dependent centroid mode for all SPS-MS3 methods. On-line chromatography was performed on a 100μm inner diameter microcapillary column packed with 35cm of Accucore C18 resin was used. Approximately 2μg of labelled peptides were loaded onto the column. Spectra were acquired across a 90-minute LC gradient ranging from 6–25% acetonitrile in 0.125% formic acid. For the 10-condition experiment, a real-time search method was employed in the instrument such that SPS-MS3 scans would not trigger unless a successful Comet search result was obtained from a preceding MS2 scan3. For the 3-condition experiment, in lieu of real-time search a field asymmetric wave form ion mobility spectrometry (FAIMS) device was attached ahead of the mass spectrometer’s source. The FAIMS was operated as described previously with 3 CV offsets set at −40, −60, and −80; SPS-MS3 scans were triggered with a traditional Top 10 protocol4. MS1 scans were obtained (Orbitrap resolution: 120,000; mass range: 400–1400 m/z; Automatic Gain Control (AGC) target = 200,000, maximum ion injection time: 100ms) prior to a low-resolution MS2 scan utilizing CID (Collision energy: 35%; maximum injection time: 35ms; AGC target: 20,000; Isolation window: 0.7 Th). Precursors from the MS1 spectra were selected for MS2 by a Top 10 method. In the case of the 10-condition experiment, MS3 scans were sent from the real-time search algorithm. For the 3-condition experiment, the SPS-MS3 scan encoded into the method was a Top 10 method (Orbitrap Resolution: 50,000; Scan Range: 100–500 m/z; maximum injection time: 120ms; AGC Target: 150,000).

### LC-MS/MS Data Analysis

Spectra from all mass spectrometry experiments were processed and searched with an in-house Sequest-based software pipeline as described previously1,5,6. Raw data acquired on mass spectrometer instruments were converted into an mzXML format before a Sequest search was executed against a human proteome database (Uniprot Database ID: 9606, downloaded February 4, 2014). This database was concatenated with common laboratory contaminants and a database comprised of all protein sequences reversed. Ion tolerances for precursor and product ions were set to 50ppm and 0.9 Da respectively. A variable mass difference of +15.99491 Da was assigned to methionine residues to account for potential oxidation. Additionally, fixed modifications on cysteine (+57.02146 Da) and lysine and the peptide N-terminus (+229.16293 Da) were assigned to account for protective alkylation and TMT-11 labelling, respectively.

The peptide level false discovery rate (FDR) was determined using the target-decoy method and controlled to 1% by linear discriminant analysis before protein level FDR correction to 1% was performed using the principles of parsimony as described previously1,6–8. Signal-to-noise ratios (SNR) for TMT reporter ions were extracted from high resolution SPS-MS3 scans. A quality control lter of 200 SNR and isolation specificity, a measure of a scan’s isolation purity, of 0.5 were imposed on the data for each experiment9. Protein level estimation for each sample was determined as the sum SNR of the appropriate TMT reporter-ion channel from all contributing peptides identified and quantified in the experiment. For the 10-condition experiment, protein level abundances were normalized across all TMT-reporter ion channels under the assumption that total protein levels were consistent across all TMT-channels before an additional normalization step ensuring that protein levels of SORL1, SUDS3, GRAP, CHMP1B, TCL1A, MECP2, ABHD10, STAP1, and PSIP1 were consistent across all TMT-channels based on RNAseq data. For the 3-condition experiment, protein level abundances were normalized across all TMT-reporter ion channels under the assumption that total protein levels were consistent across all TMT-channels.

Relative protein ratios were calculated between protein TMT reporter-ion SNR levels in a patient-matched fashion before averaging across the calculated ratios. When comparing patient samples to laboratory cell lines (in the case of the 3-conditoin experiment) protein TMT reporter-ion SNR levels were averaged before calculating a ratio. Two-factor analysis of variance was used to calculate significance to account for patient-to-patient variation. Tukey’s Honestly Significant Difference post-hoc tests were then used to identify nominal p-values between pairwise comparisons.

### Mouse xenograft experiments

Mouse xenograft experiments were done in accordance with the Institutional Animal Care & Use Committee (IACUC# 2017–0035) of Weill Cornell Medical Center (WCMC).

Twelve NOD.Cg-Prkdcscid Il2rgtm1Wjl/SzJ (NSG) immunocompromised mice were subjected to bilateral xenotransplantation with a finely minced 2–3mm^3^ piece of MCD DLBCL, HBL1 for two weeks. Once the tumor volumes reached the range between 30–60mm^3, mice were randomly divided into 3 groups. Vehicle, 5mg/kg, or 20mg/kg ERG245 was injected intraperitonially, once a week, into the mice in the three groups respectively. Tumor growth was monitored for an additional 3 weeks.

Twelve NOD.Cg-Prkdcscid Il2rgtm1Wjl/SzJ (NSG) immunocompromised mice were subjected to bilateral xenotransplantation with a finely minced 2–3mm^3^ piece of MCD DLBCL, OCI-LY10 for two weeks. Once the tumor volumes reached the range between 30–60mm^3, mice were randomly divided into 3 groups. Vehicle, 5mg/kg, or 20mg/kg ERG245 was injected intraperitonially, once a week, into the mice in the three groups respectively. Tumor growth was monitored for an additional 4 weeks.

Twelve NOD.Cg-Prkdcscid Il2rgtm1Wjl/SzJ (NSG) immunocompromised mice were subjected to bilateral xenotransplantation with a finely minced 2–3mm3 piece of patient derived MCD DLBCL tumor C007 for two weeks. Once the tumor volumes reached the range between 30–60mm^3, mice were randomly divided into 2 groups. Vehicle or 20mg/kg ERG245 was injected intraperitonially, three times a week, into the mice in the two groups respectively. Tumor growth was monitored for an additional 2 weeks.

Digital caliper measurements and body weight were measured under 2.5% isoflurane anesthesia. Two-dimensional length (L) and width (W) measurements were used to extrapolate 3D volume using the formula (L × W^2^)/2. All animals were euthanized by CO2 asphyxiation at a flow rate 3.5L/min.

### Seahorse metabolic analysis

For mitochondrial stress test analysis, cell culture plates were layered with Cell-Tak to enable B-cell adhesion, and cells were then seeded at 500,000 per well. For standard measurements, complete bicarbonate-free RPMI-1640 supplemented with 25 mM HEPES, 10% dialyzed FBS and 2 mM L-glutamine was used as the growth media during the period of data acquisition. Detection of changes in oxygen consumption and extracellular acidification rates was achieved with the use of Seahorse XFe96 sensor cartridges. The following mitochondrial poisons were used: 3.5 μM oligomycin, 2 μM CCCP and 100 nM piericidin A. Data acquisition was performed with the Seahorse XFe96 Analyzer (Agilent).

### Quantification and statistical analysis

Unless otherwise indicated, all bar graphs and line graphs represent the arithmetic mean of three independent experiments (n = 3), with error bars denoting standard deviations. Data were analyzed using two-tailed paired Student t test or analysis of variance (ANOVA) with the appropriate post-test using GraphPad Prism7 software. Gene ontology analysis was done with the Enrichr module using the KEGG pathway databases. Default parameters of Enrichr module was used, with the exception that the Enrichment statistic was set as classic. Metabolic pathway analysis were performed using MetaboAnalyst 3.0. Figures were drawn with commercially available GraphPad, Biorender, Microsoft Powerpoint.

## Data Availability

All RNA-seq datasets have been deposited to the NIH GEO omnibus. The accession number for the RNA-seq dataset reported in this paper is GSE232769. The mass spectrometry proteomics data have been deposited to the ProteomeXchange Consortium via the PRIDE partner repository with the dataset identifier PXD016961. The deposited dataset will be released upon acceptance. All plasmids and cell lines generated in this study will be made available on request.
